# Competitive Repair by Naturally Dispersed Repetitive DNA during Non-Allelic Homologous Recombination

**DOI:** 10.1371/journal.pgen.1001228

**Published:** 2010-12-02

**Authors:** Margaret L. Hoang, Frederick J. Tan, David C. Lai, Sue E. Celniker, Roger A. Hoskins, Maitreya J. Dunham, Yixian Zheng, Douglas Koshland

**Affiliations:** 1 Howard Hughes Medical Institute and Department of Embryology, Carnegie Institution, Baltimore, Maryland, United States of America; 2 Department of Biology, Johns Hopkins University, Baltimore, Maryland, United States of America; 3 Baltimore Polytechnic Institute, Ingenuity Program, Baltimore, Maryland, United States of America; 4 Life Sciences Division, Lawrence Berkeley National Laboratory, Berkeley, California, United States of America; 5 Department of Genome Sciences, University of Washington, Seattle, Washington, United States of America; The University of North Carolina at Chapel Hill, United States of America

## Abstract

Genome rearrangements often result from non-allelic homologous recombination (NAHR) between repetitive DNA elements dispersed throughout the genome. Here we systematically analyze NAHR between Ty retrotransposons using a genome-wide approach that exploits unique features of *Saccharomyces cerevisiae* purebred and *Saccharomyces cerevisiae*/*Saccharomyces bayanus* hybrid diploids. We find that DNA double-strand breaks (DSBs) induce NAHR–dependent rearrangements using Ty elements located 12 to 48 kilobases distal to the break site. This break-distal recombination (BDR) occurs frequently, even when allelic recombination can repair the break using the homolog. Robust BDR–dependent NAHR demonstrates that sequences very distal to DSBs can effectively compete with proximal sequences for repair of the break. In addition, our analysis of NAHR partner choice between Ty repeats shows that intrachromosomal Ty partners are preferred despite the abundance of potential interchromosomal Ty partners that share higher sequence identity. This competitive advantage of intrachromosomal Tys results from the relative efficiencies of different NAHR repair pathways. Finally, NAHR generates deleterious rearrangements more frequently when DSBs occur outside rather than within a Ty repeat. These findings yield insights into mechanisms of repeat-mediated genome rearrangements associated with evolution and cancer.

## Author Summary

The human genome is structurally dynamic, frequently undergoing loss, duplication, and rearrangement of large chromosome segments. These structural changes occur both in normal and in cancerous cells and are thought to cause both benign and deleterious changes in cell function. Many of these structural alterations are generated when two dispersed repeated DNA sequences at non-allelic sites recombine during non-allelic homologous recombination (NAHR). Here we study NAHR on a genome-wide scale using the experimentally tractable budding yeast as a eukaryotic model genome with its fully sequenced family of repeated DNA elements, the Ty retrotransposons. With our novel system, we simultaneously measure the effects of known recombination parameters on the frequency of NAHR to understand which parameters most influence the occurrence of rearrangements between repetitive sequences. These findings provide a basic framework for interpreting how structural changes observed in the human genome may have arisen.

## Introduction

Human structural variation contributes to phenotypic differences and susceptibility to disease [1]. Recent studies suggest that many structural variants are mediated by non-allelic homologous recombination (NAHR) between dispersed repetitive DNA elements [2]–[5]. While the importance of NAHR in shaping genome structure is becoming more apparent, the mechanism of NAHR remains poorly understood.

NAHR (also known as ectopic recombination) utilizes the molecular pathways that mediate allelic homologous recombination (AHR) between sister chromatids or homologs. AHR and NAHR are both initiated by a double-strand break (DSB) that is processed by 5′-3′ DNA resection to generate 3′-OH tailed single-stranded DNA (ssDNA) intermediates [6]. The resected ssDNA, called the recipient, is activated to search for homologous sequences, called the donor, to be used as a template for repair. If the recipient is unique DNA, then the donor will be the homolog or sister chromatid, and AHR ensues. However, if the recipient is repetitive DNA, it may choose a non-allelic repeat as a donor, leading to NAHR and potentially a chromosome rearrangement. The establishment of this basic recipient-donor partnership during homologous recombination (HR) defines four fundamental parameters for NAHR that we address here.

The first parameter is the position of a DSB relative to repetitive and unique sequences. DNA resection starts from the DSB ends and is thought to activate break-proximal sequences before break-distal sequences [7]. Based on this model, break-proximal recipients (sequences at or near the break site) direct homology searches before break-distal recipients (sequences distal from the break site). Therefore, a DSB near or in a repetitive element should activate that repeat as a recipient, which may search for a non-allelic donor repeat to promote NAHR. Alternatively, a DSB in a large track of unique sequences should preferentially activate break-proximal unique sequences as recipients. In a diploid, these break-proximal recipients can repair efficiently using allelic donors on the sister chromatid or homolog. Therefore it has been assumed, but never tested directly, that a DSB in unique sequences in a diploid will rarely induce NAHR. However, a few studies in haploid yeast have observed a preference for recombination using more distal sequences over break-proximal recipients, suggesting that break-distal recipients can participate in homology searches [8]–[10].

The second important parameter of NAHR is the percent and length of identity shared between a recipient and potential donors. Introduction of ∼1% sequence divergence between model repeats decreases recombination rates 9- to 25-fold [11], [12], suggesting that even very limited divergence may significantly affect NAHR rates. The minimum length of uninterrupted identity between two sequences needed for efficient recombination is called the minimal effective processing segment (MEPS) [13]. Using model repeats, the MEPS necessary for efficient NAHR is about 250 bp [14], [15]. This suggests that small retroelements, such as Alus (∼300 bp) and long terminal repeats (LTRs; ∼330 bp), are potentially sufficient to promote efficient NAHR. However, how homology between natural repeats relates to usage for NAHR has never been assessed at a genome-wide scale.

The third important parameter of NAHR is genomic position of a recipient and potential donors. Recipients and donors are more likely to recombine when they are on the same chromosome than when they are on different chromosomes [16]–[18]. Interchromosomal recombination between model repeats can also be influenced by their proximity to centromeres and telomeres [19], [20]. However, these NAHR position preferences have not been tested with natural repeats in an unbiased system, where the unrestricted choice of repair partners and pathways is allowed.

Finally, which HR pathway acts upon a recipient and donor may impact whether NAHR occurs. Single-strand annealing (SSA) can occur when resection from a DSB proceeds through flanking direct repeats, exposing complementary sequences that anneal to generate a deletion product [6]. In contrast, Rad51-dependent HR pathways involve strand invasion events where Rad51 polymerizes onto resected recipient DNA to mediate invasion into a homologous duplex donor. When recipient sequences on both sides of the DSB invade the same donor, repair can occur by gene conversion (GC). However, if the recipient shares identity with the donor on just one side of a DSB, then one-ended strand invasion events can repair through break-induced replication (BIR). GC is faster and more efficient at repairing DSBs than BIR [21]. In addition, GC competes effectively with SSA [22], [23]. While the competition between SSA, GC, and BIR can influence NAHR outcomes, little is known about the relative usage of these pathways during NAHR with natural repeats.

Thus the efficiency and outcome of NAHR are potentially influenced by its ability to compete with AHR, the sequence identity between recipients and donors and their genomic position, and the usage of HR pathways. Yet these potential influences remain untested or unresolved, particularly in the context of a family of naturally repeated sequences. To address these fundamental issues, we developed a new genome-wide system to study NAHR between the dispersed and divergent families of Ty retrotransposons in purebred and hybrid diploids of budding yeasts. We exploit this system to provide insight into the most important parameters that control NAHR in a eukaryotic diploid genome.

## Results

### A genome-wide system to study NAHR events between Ty1/Ty2 families of repeats

Ty1 and Ty2 represent the most abundant families of dispersed repetitive elements in *S. cerevisiae*. Our system to study Ty-mediated NAHR relies on three components: (1) knowledge of the sequence and position of all Ty1/Ty2 elements in the genome, (2) strains with genetic features for the recovery of Ty-mediated NAHR events, and (3) a protocol to measure these events out of all possible outcomes. Below we provide a brief description of each component.

**Figure 1:**
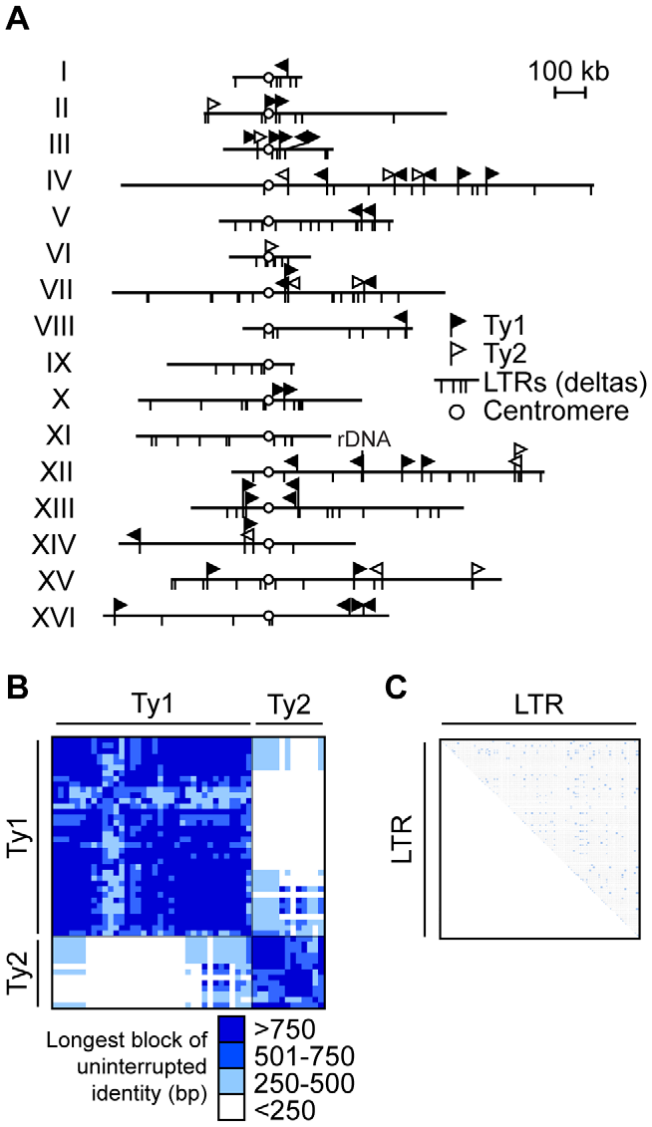
Ty retrotransposon elements in *S. cerevisiae* are tractable repetitive families to study non-allelic homologous recombination (NAHR). (A) Diagram showing the insertion sites for Ty1/Ty2 families of long terminal repeats (LTR) retrotransposons on *S. cerevisiae* sixteen chromosomes, aligned by their centromeres (white circle). Insertion sites for full length Ty1 (black flag) and Ty2 (white flag) are shown as perpendicular lines above chromosomes while insertion sites for solo Ty1/Ty2 LTRs (also called deltas) are perpendicular lines below chromosomes. Continuous lines above and below chromosome show the same insertion site for full length Tys and solo LTRs. Ty1 and Ty2 are the most abundant of the Ty families and closely related, sharing almost identical LTR sequences. Note that the diagram is drawn to scale except for chromosome XII where 1–2 Mb rDNA array position is noted. Heat map representing the longest block of uninterrupted identity of pairwise comparisons between 50 Ty1/Ty2 in (B) and 306 LTRs (C) in the *S. cerevisiae* genome. Length of blocks are binned and colored in intervals of 250 base pairs (bp), as indicated in key below. Binning analysis based on MEPS of ∼250 bp, the minimal length of identity empirically determined for efficient NAHR in yeast [15]. Note that most comparisons are above this MEPS value (blue-shaded), predicting that the majority of Ty1/Ty2 pairings are competent for efficient NAHR. Details for (B) in Table S4 and (C) in Table S5.

As a first step, we completed the sequence of the *S. cerevisiae* unannotated chromosome III Ty elements (Figure S1). With the completed sequence, we generated a map of the distribution of 37 full length Ty1s and 13 full length Ty2s [which includes 98 Ty-associated 5′ and 3′ long terminal repeats (LTRs)], and 208 solo LTRs (Figure 1A). The sequence and positional information is critical since it defines all potential Ty1/Ty2 recipients and donors in the *S. cerevisiae* genome, allowing us to determine whether some repeats are used and others are not in NAHR.

The potential for Ty elements to act as recipients and donors in NAHR depends in part on their sequence identity. The average percent sequence identity is 95.7±2.4% between Ty1s, 95.9±4.8% between Ty2s, and 73.9±3.4% between Ty1 and Ty2 (Table S3). Previous work has determined that recombination between model repeats decreases 9-fold with 99% identity and 50-fold with 91–94% identity relative to identical model repeats [11]. Thus the sequence divergence of the Ty1/Ty2 family could dramatically reduce the pool of potential Ty recipients and donors, limiting the number of elements that participate in NAHR.

However, if the mismatches are clustered, rather than distributed evenly within the full length of Ty1/Ty2 (5.9 kb), then long stretches of identity may allow efficient NAHR. With this in mind, we analyzed the longest block of uninterrupted identity between all pairwise alignments of Ty1/Ty2, a parameter that has not been previously assessed for Ty elements. To evaluate the significance of these blocks, we categorized them according to the previously determined MEPS value of about 250 bp for NAHR [14], [15]. Recombination rates are predicted to significantly drop when lengths are below MEPS and proportionally increase when lengths are above MEPS [13].

Using our binning analysis, 73% of all Ty1/Ty2 alignments (891 out of 1225) have blocks of identity ≥250 bp (Figure 1B and Table S4). All pairwise comparisons between repeats within either the Ty1 or Ty2 family are above the MEPS value while 31% of pairwise comparisons between Ty1 and Ty2 repeats have a block of identity ≥250 bp. Thus, for the full length Ty1s and Ty2s, the shared blocks of uninterrupted identity strongly predict that a given Ty1/Ty2 recipient can undergo NAHR with many potential Ty1/Ty2 donors, thereby establishing a competition among donors. In contrast, only 1% of all LTR pairwise comparisons (544 out of 46,665) have a block of uninterrupted identity ≥250 bp (Figure 1C and Table S5). This limited length of uninterrupted identity between the LTRs predicts that they may be inefficient substrates for NAHR. In addition, sequence identity amongst pairwise comparisons of the 306 LTR elements widely range between 3%–100%, with an average of 59.6%±22.7% (Table S6). Thus the poor sequence identity between LTRs suggests that solo LTRs will be unfavorable substrates for NAHR.

**Figure 2:**
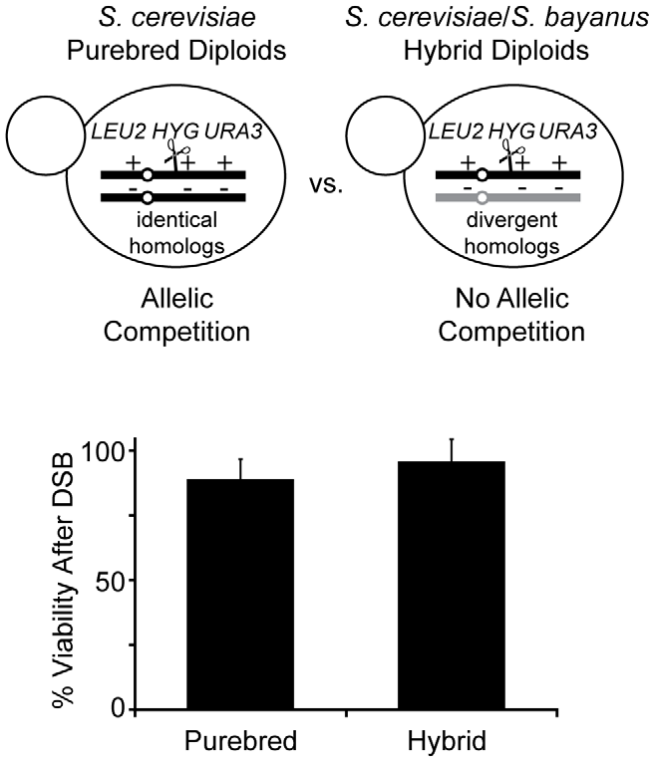
Diploids allow recovery of all possible NAHR events without loss of viability. Top: NAHR between *S. cerevisiae* Ty elements may be determined with or without allelic competition in *S. cerevisiae* purebred and *S. cerevisiae*/*S. bayanus* hybrid diploids, respectively. *LEU2*, *HYG*, *URA3* are heterozygous in both diploids to classify events that occur on the *S. cerevisiae* chromosome III homolog containing the I-*Sce*I cut site (cs, scissors). For more detail, see Figure 3 below. Bottom: Viability after a DSB in hybrid diploids (MH3360) and purebred diploids (MH3359). Relative viability assayed by colony forming units (CFUs) on –ade +2% galactose plates (continuous induction of I-*Sce*I-induced DSB) compared to CFUs on –ade +2% glucose plates (no induction of DSB). Error bars indicate SD performed on four independent experiments.

The second component of our system is the use of specific strains to optimize the recovery of Ty-mediated NAHR events. In order to recover all possible NAHR events, we use diploid yeast where loss of genetic material can be complemented by homologs. In contrast, Ty-mediated rearrangements that occur in haploids may delete genes necessary for viability. Along with *S. cerevisiae* diploids (referred hereafter as “purebred”), we generated synthetic hybrid diploids by mating *S. cerevisiae* with a sequenced relative, *S. bayanus* (referred hereafter as “hybrid”) (Figure 2), which is largely devoid of Ty1/Ty2 elements [24], [25]. The diploids are genetically marked to allow identification of all cells that suffer an I-*Sce*I site-specific DSB as well as the subset of cells in which the broken chromosome is repaired or lost (Figure 2 and see below). Like the purebreds, viability remains high after induction of an I-*Sce*I-induced DSB in the hybrid diploids (Figure 2). In addition, the hybrid diploids grow well and are competent in DNA maintenance and repair like the purebred diploids (Figure S2). Since *S. bayanus* complements almost all the genes in *S. cerevisiae* [26], *S. bayanus* can also balance *S. cerevisiae* by suppressing any loss of gene function due to NAHR of the *S. cerevisiae* genome. However, in contrast to the purebred diploids, the hybrid diploids have three important advantages. The significant sequence divergence between the two genomes (62% intergenic, 80% genic) [27] suppresses AHR, favoring NAHR between the more homologous Ty1/Ty2 elements and thus enhancing the recovery of Ty-mediated NAHR events. The sequence divergence also facilitates the analysis of *S. cerevisiae* rearrangements by array comparative genomic hybridization (aCGH) and PCR. Finally, the comparison of NAHR between the purebred and hybrid diploids allows the assessment of NAHR with and without AHR competition (Figure 2).

**Figure 3:**
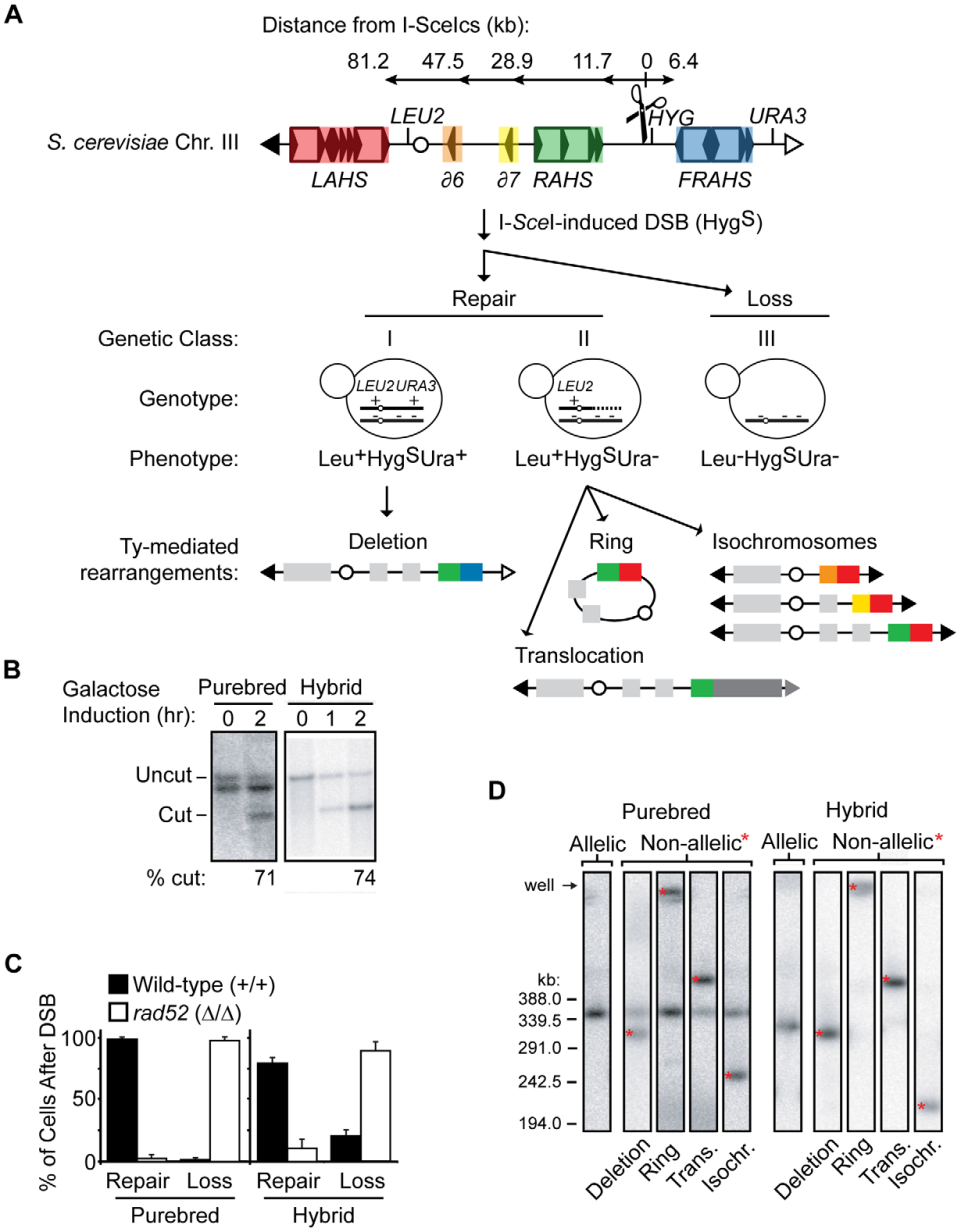
Nonselective assay to determine frequency of NAHR out of all possible outcomes after a DSB. (A) Flow chart showing the nonselective characterization of clones after galactose induction of I-*Sce*I endonuclease (see text for details). Top: Map of *S. cerevisiae* chromosome III showing the I-*Sce*Ics (scissors) at position 163cs (number refers to chromosome III SGD coordinates in kb); centromere, white circle; left telomere, black triangle; right telomere, white triangle. Tys are represented as open rectangles flanked by solid triangles (LTRs). Five Ty insertion loci are highlighted in red, left arm transposition hotspot (*LAHS*); orange, *YCRCdelta6* (*∂6*); yellow, *YCRCdelta7* (*∂7*); green, right arm transposition hotspot (*RAHS*); blue, far right arm transposition hotspot (*FRAHS*) (see Figure S1 for more detail of Ty elements). Bottom: Clones were scored for heterozygous genetic markers (*LEU2, HYG, URA3*) to determine whether the founding cell had experienced an I-*Sce*I-induced DSB (Hygromycin-sensitive, Hyg^S^) followed by repair (class I, Leu^+^Hyg^S^Ura^+^; class II, Leu^+^Hyg^S^Ura^−^) or loss of the broken chromosome (class III, Leu^−^Hyg^S^Ura^−^). Ty-mediated NAHR rearrangement structures from these repair classes (details in Materials and Methods) show recipient-donor partners at the recombination junctions according to Ty locus color in map above. (B) Southern blots showing that the majority of cells initiate the I-*Sce*I site-specific DSB at 163cs after galactose induction in purebred (MH3359) and hybrid (MH3360) diploids. *YCR024C* probe also hybridizes to the other homolog in purebred diploids (smaller size than uncut band due to the absence of the 1.6 kb I-*Sce*Ics/*HYG* construct). (C) Frequencies of *S. cerevisiae* chromosome III repair (class I+II) or loss (class III) after DSB in wild-type (MH3359) and *rad52* (MH3475) purebred diploids, and wild-type (MH3360) and *rad52* (MH3476) hybrid diploids. Error bars indicate SD. At least two independent experiments assayed for each strain. (D) PFGE/Southern analysis on representative repair clones in purebred (MH3359) and hybrid (MH3360) diploids. Note that, in purebred diploids, the uncut homolog contains *leu2Δ1* allele which also hybridizes with the *LEU2* probe and, in hybrid diploids, allelic HR occurs at a low frequency between the divergent homologs.

The third component of our system is an unbiased clone-based assay to determine the frequencies of NAHR events among all possible outcomes (Figure 3A). An I-*Sce*I recognition sequence [referred to as the I-*Sce*I cut site (cs)], along with a Hygromycin-resistance gene (*HYG*), is integrated at different positions on the *S. cerevisiae* chromosome III homolog. We choose to initiate a DSB on the *S. cerevisiae* chromosome III since this chromosome has the highest density of Ty1/Ty2 elements relative to all other chromosomes (see Figure 1A), making it a good model for the repetitive-rich chromosomes of higher eukaryotes. We initiate the DSB with the addition of galactose to the media for two hours in exponentially growing cultures to induce expression of the I-*Sce*I endonuclease fused to the galactose promoter. Galactose induction of I-*Sce*I expression leads to formation of a DSB at the 163cs position on one *S. cerevisiae* chromosome III homolog (Figure 3B), which activates recipient sequences adjacent to the break site to undergo a homology search. The cells are then plated onto nonselective YEPD media for individual colonies (referred to as clones). These clones are then phenotyped to determine whether the I-*Sce*I-induced DSB occurred (Hyg^S^, see Figure S3) followed by chromosome repair (Leu^+^Ura^+^ or Leu^+^Ura^−^) or loss (Leu^−^Ura^−^). We find that the majority of I-*Sce*I-induced DSBs are repaired in both the purebred (99±2%) and hybrid (79±5%) diploids, although the hybrid diploids exhibit a significant increase in chromosome loss (20±5%) compared to the purebred diploids (1±2%) (Figure 3C). HR mediates almost all of this DSB repair in both diploids since repair is nearly abolished when the essential HR protein Rad52 is absent (Figure 3C).

To assess the structure of the repaired chromosome in the two genetic repair classes, a random subset of clones in each class are further analyzed by pulse-field gel electrophoresis (PFGE)/Southern analysis (Figure 3D). An I-*Sce*I-induced DSB at the 163cs position that is repaired by AHR results in an unchanged chromosome III size whereas repair by NAHR results in a rearrangement with a changed chromosome III size (Figure 3D). Further aCGH and PCR characterization of the genetic repair classes reveals four types of chromosome III rearrangement structures with Ty elements localized to the recombination junctions (Figure S4 and see Materials and Methods). The Leu^+^Hyg^S^Ura^+^ repair class I contains internal deletions, and the Leu^+^Hyg^S^Ura^−^ repair class II includes isochromosomes, rings, and translocations (see schematics in Figure 3A). The recovery of these distinct Ty-mediated NAHR rearrangements from one site-specific DSB reveals a competition between recipient and donor Ty elements for NAHR, validating our system as a means to study NAHR between complex families of natural repeats.

### Recipient competition revealed by break-distal recombination (BDR)

A site-specific DSB in unique DNA allows us to assess the likelihood that break-distal repeats are activated as recipients in a homology search to facilitate NAHR. HR events that use a break-distal recipient for recombination are termed here as break-distal recombination (BDR). With 163cs positioned inside 18.1 kb of unique DNA on chromosome III (see map in Figure 3A), we tested the possibility for BDR by monitoring three potential Ty recipient loci (*YCRCdelta6*, *YCRCdelta7*, *RAHS*) at various distances distal from the break site. Because our assay employs no selection, we are able to calculate the frequencies of I-*Sce*I-induced Ty-mediated rearrangements among all possible outcomes after the DSB (see Materials and Methods). Below we highlight the major points from the data compiled in Table 1 and Table 2.

**Table 1:** Frequencies of outcomes after an I-*Sce*I-induced DSB at 163cs* on *S. cerevisiae* chromosome III in wild-type and recombination mutants.

			*n* (number of clones)			Frequency of Outcome After DSB (% ± SEM)^a^						
			Total^b^	Repair^c^		NAHR events^d^						
Diploid Type^e^	Strain	Geno-type^f^	Hyg^S^	Class I	Class II	Int. Del.	Iso-chr.^g^	Ring	Trans.	Other^h^	Al-lelic^i^	Chr. Loss
Hybrid	MH3360	WT	955	18	52	60.6±3.3	10.8±1.3^j^	3.2±1.0	1.4±0.7	0.4±3.6	2.9±4.2	20.7±2.4
	MH3476	*rad52*	287	17	0	11.2±0.6	<0.3	<0.3	<0.3	<0.6	<0.6	88.8±7.1
	MH3726	*rad59*	1253	14	26	54±3.7	4.5±0.8	1.9±0.7	1.0±0.5	0.6±4.2	0.3±4.1	37.3±3.0
	MH3507	*rad51*	502	22	17	69.8±3.1	3.6±0.2	<0.2	0.2±0.2	<3.3	<3.3	26.3±2.7
	MH3699	*msh2*	975	18	30	74.1±4.0	3.2±0.6	1.3±0.5	1.1±0.4	0.2±4.2	0.6±4.4	19.5±1.8
	MH3692	*msh6*	2629	17	29	55.1±3.1	17.3±3.1	10.4±2.9	3.5±1.9	<4.3	2.3±4.7	11.4±0.7
	MH3455	*sgs1*	644	34	49	55.1±1.6	15.0±2.5	11.4±2.3	4.3±1.6	<2.3	4.3±3.2	10.0±2.9
Purebred	MH3359	WT	1062	32	46	13.4±5.5	3.2±0.8	0.3±0.3	0.3±0.3	0.3±2.9	81.5±6.4	1.0±1.0
	MH3475	*rad52*	227	8	0	5.0±0.7	<0.4	<0.4	<0.4	<1.0	<1.0	93.9±2.4
	MH3502	*rad51*	258	24	20	58.7±2.4	7.6±1.2	<0.6	1.8±0.9	2.3±3.4	<3.6	55.1±1.6

*I-*Sce*I cut site/*HYG* construct inserted at SGD coo. 163491; DSB is 11,657 bp from *RAHS*, 28,874 from *YCRCdelta7*, and 47,488 bp from *YCRCdelta6*, map in Figure 3A.

a Total frequencies of outcomes are normalized to 100%.

b Total number of clones after galactose induction that suffered a DSB (Hyg^S^) and were scored for chromosome repair or loss.

c Random repair clones from Class I (Leu^+^Hyg^S^Ura^+^) and Class II (Leu^+^Hyg^S^Ura^−^) that were analyzed by PFGE/Southern analysis.

d Rearrangements are diagrammed in Figure 3A, except Other.

e Hybrids are *S. cerevisiae* (*MATα*) crossed with *S. bayanus* (*MATa*). Purebreds are *S. cerevisiae* (*MATα*) crossed with *S. cerevisiae* (*MATa*).

f Relevant genotype noted, see Table S1 for full genotype.

g Three different recipients (*YCRCdelta6*, *YCRCdelta7*, *RAHS*) mediate isochromosomes with the *LAHS* donor.

h Other refers to repair clones that were from an uncharacterized size category observed by PFGE/Southern analysis.

i In hybrid diploids, recombination between the divergent homologs results in a *S. bayanus* chromosome III size product (∼310 kb). This was assigned as allelic in hybrids.

j 6.5% mediated by *YCRCdelta6*, 1.8% by *YCRCdelta7*, 2.5% by *RAHS.*

DSB, double-strand break; Hyg^S^, Hygromycin-sensitivity; SEM, standard error of the mean; NAHR, non-allelic homologous recombination; Int. Del., internal deletion; Isochr., isochromosome; Trans., translocation; Chr. loss, chromosome loss; WT, wild-type.

**Table 2:** Frequencies of outcomes after an I-*Sce*I-induced DSB with different *S. cerevisiae* chromosome III configurations.

				*n* (number of clones)			Frequencies of Outcomes After DSB (% ± SEM)^a^								
				Total^b^	Repair^c^		NAHR events^d^								
Di-ploid Type^e^	Strain	I-SceI cut site	Ty locus deletion^f^	Hyg^s^	Class I	Class II	Ty GC^g^	Intra-Ty Del.^h^	Inter-Ty Del.^i^	Iso-chr.^j^	Ring	Trans.	Other^k^	Allelic^l^	Chr. Loss
Hy-brid	MH3360#	163cs^m^	None	955	18	52	n.a.	n.a.	60.6±3.3	10.8±1.3^n^	3.2±1.0	1.4±0.7	0.4±3.6	2.9±4.2	20.7±2.4
	*	163cs	*FRAHSΔ*	503	5	19	n.a.	n.a.	n.a.	12.7±3.1	11.3±3.0	<1.4	<2.0	6.0±2.4	70.1±3.4
	MH3398	163cs	*LAHSΔ*	437	18	11	n.a.	n.a.	70.1±3.8	n.a.	n.a.	1.5±0.5	0.3±4.1	1.5±4.3	26.5^o^
	MH3551	151cs^p^	*FRAHSΔ*	589	5	48	n.a.	n.a.	n.a.	27.5±2.8	9.4±2.5	1.7±1.2	2.9±1.4	0.9±1.1	57.6±1.9
	MH3768	RAHScs^q^	None	350	24	18	22.1±7.8	59.1±8.5	7.4±5.0	3.7±0.6	1.5±0.6	0.3±0.3	<3.9	<3.9	5.8±0.2
	MH3471	147cs^r^	None	723	4	22	n.a.	n.a.	n.a.	5.9±0.9	n.a.	<0.4	1.6±1.0	2.7±1.2	89.8±1.0
Pure-bred	MH3359#	163cs	None	1062	32	46	n.a.	n.a.	13.4±5.5	3.2±0.8	0.3±0.3	0.3±0.3	0.3±2.9	81.5±6.4	1.0±1.0
	MH3469	147cs	None	1124	6	6	n.a.	n.a.	n.a.	2.8±2.6	n.a.	<2.6	5.6±14.2	86.7±13.9	4.9±0.9
	MH3764	RAHScs	None	801	24	24	33.2±9.5^s^	61.3±8.8	<3.7	1.5±0.5	0.8±0.4	0.5±0.3	0.8±4.2	n.d.	1.9±0.4
	MH3523	163cs	*FRAHSΔ*	1565	36	27	n.a.	n.a.	n.a.	0.5±0.2	0.2±0.1	0.1±0.1	0.2±0.1	98.4±0.2	1.1±0.6

# Data from Table 1, repeated here for convenient reference.

**a–e, j–l,n:** See Table 1 for details.

f *S. cerevisiae* chromosome III with wild-type Ty configuration is designated as “None”.

g Ty gene conversion (GC) events have a Leu^+^Hyg^S^Ura^+^ phenotype with a wild-type *S. cerevisiae* chromosome III size. However, a small fraction of Ty GC events in purebreds have a Leu^+^Hyg^S^Ura^−^ phenotype (see ^s^), most likely the result of gene conversion with crossover. In hybrids, Ty GC events are NAHR events since repair must use a non-allelic Ty donor from *S. cerevisiae* genome. In purebreds, we cannot distinguish if Ty GC events are through NAHR since an allelic or non-allelic Ty donor may be used. However, it is likely that the majority of Ty GC repairs using an intrachromosomal Ty donor (Roeder *et al*., 1984), making these NAHR events.

h Deletion (Del.) contained within *RAHS* locus (“intra”); DSB is flanked by 1,571 bp of perfect identity in *RAHS* cluster.

i Deletion between two different Ty loci (“inter”), *RAHS* and *FRAHS*; labeled “inter-Ty” here to distinguish between intra-Ty deletions; inter-Ty is the same as internal deletions in Table 1.

m I-*Sce*I cut site/*HYG* construct inserted at SGD coo. 163491; DSB is 11,657 bp from *RAHS*, 28,874 from *YCRCdelta7*, and 47,488 bp from *YCRCdelta6*.

o No Loss SEM, genetic analysis from one experiment.

p I-*Sce*I cut site/*HYG* construct inserted at SGD coo. 151931; DSB is 97 bp from *RAHS*, 17,314 bp from *YCRCdelta7*, and 35,929 bp from *YCRCdelta6*.

q I-*Sce*I cut site/*HYG* construct inserted in position 5310 in *RAHS* Genbank accession GU220389 (TyB-POL of *YCRWTy1-2*).

r I-*Sce*I cut site/*HYG* construct inserted at SGD coo. 147932; DSB is 4,869 bp from *YCRCdelta7* and 23,483 bp from *YCRCdelta6*

s 30.6±8.8 are Leu^+^Hyg^S^Ura^+^ and 2.6±0.6 are Leu^+^Hyg^S^Ura^−^.

*Compiled from strain MH3524, MH3572, MH3573; same strain transformed with different I-*Sce*I plasmids (see Table 1).

n.a., not applicable. Ty locus deletion(s) result in the absence of the corresponding rearrangement size class by PFGE/Southern analysis. Ty GC and Intra-Ty events occur with DSB in repetitive sequences at RAHScs, but not with DSB in unique sequences (147cs, 151cs, 163cs).

n.d., not determined in purebreds since cannot distinguish between allelic or non-allelic Ty donor for Ty GC category.

DSB, double-strand break; SEM, standard error of the mean; NAHR, non-allelic homologous recombination; Isochr., isochromosome; Trans., translocation; Chr. loss, chromosome loss; cs, cut site; *LAHS*, left arm transposition hotspot; *RAHS*, right arm transposition hotspot; *FRAHS*, far right arm transposition hotspot.

**Figure 4:**
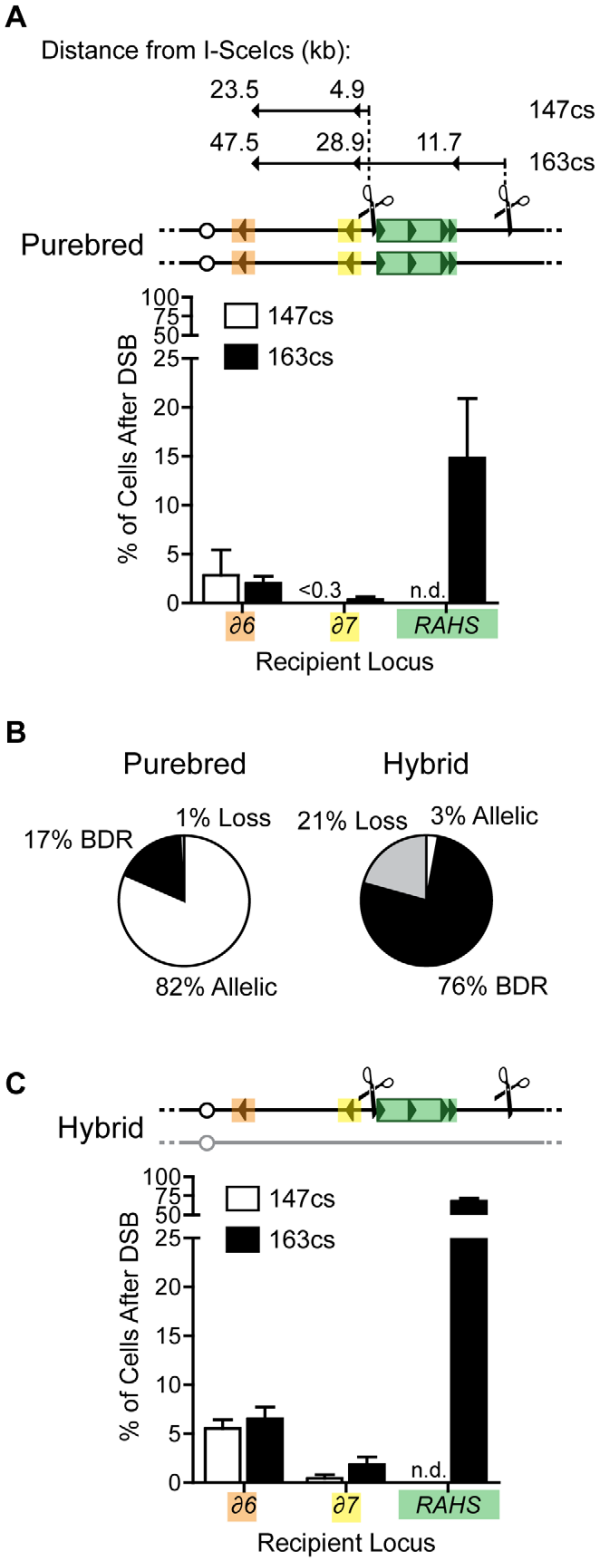
Recipient competition: Ty elements distal from DSB mediate break-distal recombination (BDR) and compete with AHR. (A) Top: Map indicating the distance of Ty recipients (orange, *δ6*; yellow, *δ7*; green, *RAHS*) from the I-*Sce*Ics at two different positions, 147cs and 163cs. The distance indicates the minimal distance from a DSB that recipient sequences are used for recombination. Bottom: Frequencies of *δ6*, *δ7*, and *RAHS* recipients localized to recombination junctions of Ty-mediated rearrangements (BDR events) in purebred diploids with a DSB at 147cs (MH3469) and 163cs (MH3359). (B) Frequencies of outcomes after a DSB at 163cs in purebred (MH3359) and hybrid (MH3360) diploids. (C) Frequencies of *δ6*, *δ7*, and *RAHS* recipients localized to recombination junctions of Ty-mediated rearrangements (BDR events) in hybrid diploids with a DSB at 147cs (MH3471) and 163cs (MH3360). Note that *RAHS* and *FRAHS* generate internal deletions through single-strand annealing (SSA, see text below) and the *RAHS* assignment of “recipient” for these rearrangements aids in comparisons. Error bars indicate SEM.

In purebred diploids, 17% of cells after DSB at 163cs undergo NAHR through BDR to mediate rearrangements. Despite a sufficient length of unique sequences that can facilitate AHR with the identical homolog after the DSB, 15±6% of cells use the *RAHS* recipient, 0.3±0.3% of cells use *YCRCdelta7*, and 2±0.7% of cells use the *YCRCdelta6* recipient located 11.7 kb, 28.9 kb, and 47.5 kb distal from the DSB, respectively (Figure 4A). To test the robustness of BDR, we changed a number of parameters. We eliminated the nonhomology immediately at the DSB ends (1.6 kb I-*Sce*Ics/*HYG* construct) to test whether BDR is due to the presence of nonhomologous ends, which may inhibit the coordination of two-ended strand invasion events during GC [28]. However, with identity at the DSB ends, BDR is still observed, generating rearrangements (Figure S5). We further tested if BDR was specific to the 163cs position by moving the position of the DSB more centromere-proximal. With the I-*Sce*I-induced DSB at 147cs, BDR-mediated Ty rearrangements occur in 3±3% of cells after DSB (Figure 4A). Interestingly, the frequency of *YCRCdelta6/YCRCdelta7* usage is similar to when a DSB initiates at 163cs, suggesting that the usage of these LTR recipients is not determined by their distance from the break site. Lastly, we tested if BDR occurs when the I-*Sce*I-induced DSB initiates on a different chromosome. BDR still occurs in 8±4% of cells after formation of a DSB on *S. cerevisiae* chromosome V to generate Ty-mediated rearrangements (Figure S6). Thus distal repeats mediate BDR despite the presence of break-proximal unique DNA that can effectively facilitate AHR. This result suggests that unique and repetitive recipient sequences at least 47.5 kb distal to a DSB can participate in recombination.

To test whether AHR competes with BDR, we analyzed BDR in the hybrid diploids. In the hybrid diploids, AHR is mostly suppressed compared to purebred diploids (3±4% of cells after DSB in hybrid compared to 82±6% of cells after DSB in purebred, Figure 4B), as expected from the extent of divergence between *S. cerevisiae* and *S. bayanus* genomes. Under these conditions of suppressed AHR, the frequency of BDR increases 4.5-fold compared to purebred diploids (increasing from 17% to 76%, Figure 4B), indicating that BDR competes with AHR. Furthermore, the distribution of different BDR-mediated rearrangements remains the same between hybrid and purebred diploids (compare Figure 4C to Figure 4A, and Table 1). Thus the presence of a divergent homolog at the break site enhances BDR-mediated rearrangements but does not alter preferences of Ty recipient and donors on chromosome III. This aspect of hybrid diploids makes them an excellent model to investigate the features of the recipients and donors that give rise to their preferred use.

To begin to define the parameters that influence the preferred use of recipient sequences to repair a DSB, we determined the largest block of uninterrupted identity between the recipient and its donor. The DSB at 163cs is positioned in the right arm of chromosome III distanced 57.4 kb from the centromere and 165.6 kb from the right telomere. Thus for AHR in purebred diploids, there is >50 kb of identity with the homolog on both sides of the DSB. In contrast, among the BDR events, the largest block of uninterrupted identity with the donors is 1,877 bp for the *RAHS* recipient, 29 bp for *YCRCdelta7* recipient, and 98 bp for the *YCRCdelta6* recipient. This reveals that the homology search in purebred diploids can be efficiently directed by 0.1%, 0.2%, or 3% (29, 98 or 1,877 bp out of 57,453 bp) of the potential recipient sequences activated by the DSB, and that this small fraction very distal to the break site generates rearrangements in a total of 17% of cells after DSB. In addition, the smaller and more break-distal solo LTRs, *YCRCdelta6* and *YCRCdelta7*, compete effectively with the larger and more break-proximal *RAHS* cluster in both purebred and hybrid diploids (see Figure 4A and Figure 4C). These data are consistent with our analysis of AHR in hybrid diploids, where the recombinant junctions occur both proximal and distal to the break site (data not shown). Moreover, these hybrid allelic junctions do not coincide with the longest length of uninterrupted identity (138 bp) found between potential recipients through *S. cerevisiae* and *S. bayanus* chromosome III alignments. Thus the relative effectiveness of repetitive and unique recipient sequences competing next to the DSB is not solely predicted by length of uninterrupted identity or distance from the DSB.

### Donor competition dictated by an intrachromosomal position bias

**Figure 5:**
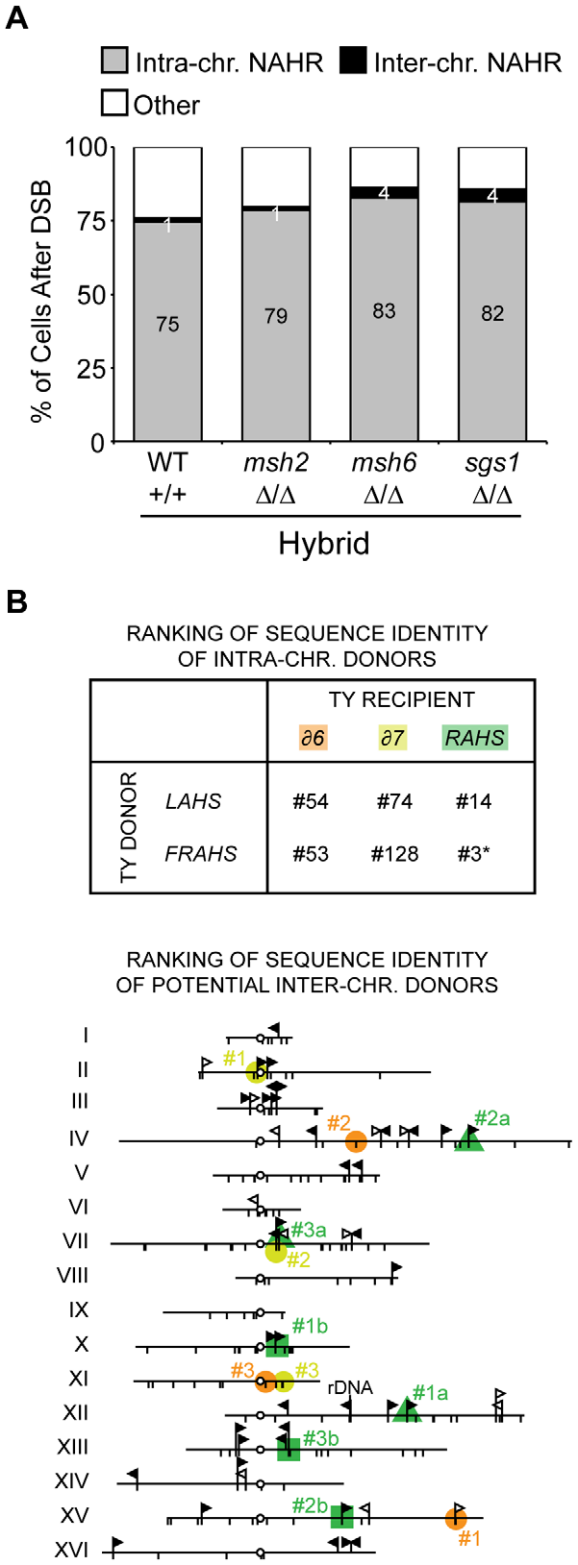
Donor competition: primary determinant is genomic position, not Ty sequence homology. (A) Frequencies of intra- and interchromosomal NAHR events after a DSB at 163cs in wild-type (MH3360), *msh2* (MH3699), *msh6* (MH3692), *sgs1* (MH3455) hybrid diploids. Internal deletions, isochromosomes, and rings are ‘Intra-chr. NAHR’, translocations are ‘Inter-chr. NAHR’, and remaining outcomes (allelic and loss) are ‘Other’. Percentages for Inter-chr. NAHR and Intra-chr. NAHR indicated in white and black, respectively. (B) Top: Ranking of sequence identity of chromosome III recipients (*δ6*, *δ7*, and *RAHS*) with intrachromosomal donors (*LAHS* and *FRAHS*) out of all donors in the *S. cerevisiae* genome (out of 305 LTRs for *δ6* and *δ7*, out of 49 Ty1/Ty2 for *RAHS*). Since multiple Ty elements are present at *RAHS*, *LAHS*, and *FRAHS*, only the highest ranking through local identity (BLAST) comparisons are indicated. *#3 donor ranking for *RAHS* recipient is attributed to the oppositely oriented *YCRCTy1-4* at *FRAHS.* However, *YCRCTy1-5* (#15 out of 49) at *FRAHS* likely mediates internal deletions due to its direct orientation with *RAHS*. Bottom: Position of the top three potential interchromosomal donors (#1–3 based on local identity) with chromosome III recipients (orange, yellow, and green correspond to *δ6*, *δ7*, and *RAHS* recipients, respectively). Since *YCRWTy1-2* and *YCRWTy1-3* are both present at *RAHS*, top three potential interchromosomal donors for each are indicated as #1a–#3a (green triangle) and #1b–#3b (green square), respectively. Symbols are as Figure 1A. Details of the ranking lists are in Table S7 and Table S8.

Our characterization of Ty-mediated NAHR events also allowed us to investigate the preferred usage of Ty donors with a DSB at 163cs. Intrachromosomal Ty sequences are used as donors in 75±4% of hybrid and 17±6% of purebred cells after DSB at 163cs, generating internal deletions, isochromosomes, or chromosome rings (intra-NAHR in Figure 5A and Table 1). In contrast, only 1±0.7% and 0.3±0.3% of cells after DSB at 163cs produce Ty-mediated interchromosomal translocations in hybrid and purebred diploids, respectively (inter-NAHR in Figure 5A and Table 1). Thus despite the greater number of potential inter- than intrachromosomal Ty donors (see Figure 1A), Ty donors on the same chromosome are preferred approximately 50 times more than Ty donors on a different chromosome.

Again as a first assessment, we wondered whether the NAHR biases for intra- over interchromosomal donors and amongst the two intrachromosomal donors (*LAHS* and *FRAHS*) are dictated by sequence identity between the donors with its Ty recipient. We generated a ranked list of sequence homology, comparing the three Ty recipient elements distal to 163cs (*YCRCdelta6, YCRCdelta7, RAHS*) with all potential Ty donor elements in the genome. We find that the intrachromosomal Ty donors (*LAHS* and *FRAHS*) are not among the most identical by either percent sequence identity or the longest block of uninterrupted identity (Figure 5B and Table S7, Table S8). Of the intra-NAHR Ty partners, we also find no correlation with the extent of sequence homology between the chosen Ty donors and their frequency of usage. For example, in the hybrid diploids, 61±3% of cells after DSB generate internal deletions between *RAHS* and *YCRWTy1-5* at *FRAHS* (97% identity, 1,635 bp largest block of uninterrupted identity) whereas only 3±1% of cells after DSB generate a chromosome ring between the same *RAHS* recipient and the *LAHS* donor (97% identity, 1,877 bp largest block of uninterrupted identity). Furthermore, relaxing the stringency for sequence identity in NAHR using *msh2Δ/msh2Δ*, *msh6Δ/msh6Δ*, and *sgs1Δ/sgs1Δ* mutants in hybrid diploids does not abolish the intrachromosomal donor preference (Figure 5A), further suggesting that the preferred usage of donors is not due to sequence identity [29], but donor position. Similar to the findings for the usage of recipient sequences for NAHR, the preferred usage of Ty donors is neither dictated nor can be predicted by sequence homology. Thus the primary determinant of Ty donor choice during NAHR is genomic position, with ∼50-fold preference for intrachromosomal over interchromosomal donors.

### Intrachromosomal position effect is due to the inefficiencies of NAHR pathways

Sequence homology between the Ty1/Ty2 families failed to dictate the recipient and donor competition during NAHR. One explanation is that each Ty-mediated rearrangement requires different genetic factors (Table 1), suggesting that they are generated through distinct NAHR pathways. Since HR pathways are known to compete after a DSB, we examined how this competition affected recipient and donor choice. In the hybrid diploids with the I-*Sce*I-induced DSB in unique sequences at 163cs, 61±3% of cells form internal deletions between the *RAHS* recipient and the *FRAHS* donor (Table 1). These deletions form independent of *RAD51* suggesting they occur through SSA (Table 1). *RAHS* also mediates isochromosomes (3±1%) and rings (3±1%) with the *LAHS* donor, and translocations with interchromosomal Ty donors (1±0.7%), all of which have Rad51-dependencies (Table 1). Thus the same *RAHS* recipient mediates internal deletions 20–40 fold higher than isochromosomes, rings, or translocations, suggesting that SSA dominates the NAHR pathway choice to generate Ty-mediated rearrangements when a DSB occurs in unique sequences.

With at least four NAHR pathways operating after the DSB at 163cs (suggested by the different genetic dependencies of the Ty-mediated BDR rearrangements, see Table 1), we then asked if these NAHR pathways were in competition with one another. To address pathway competition, we attempted to abolish or enhance particular NAHR pathways by removing their intrachromosomal donors and/or repositioning the I-*Sce*Ics in the hybrid diploids. We then compared changes in the frequencies of the Ty-mediated rearrangement product as a readout of their NAHR pathway, where compensatory effects indicate competing pathways. In addition, since Rad51-independent SSA and Rad51-dependent pathways have been shown to compensate for each other after a DSB and hence compete [22], [30], our analysis groups the NAHR pathways into these two distinct HR mechanisms.

**Figure 6:**
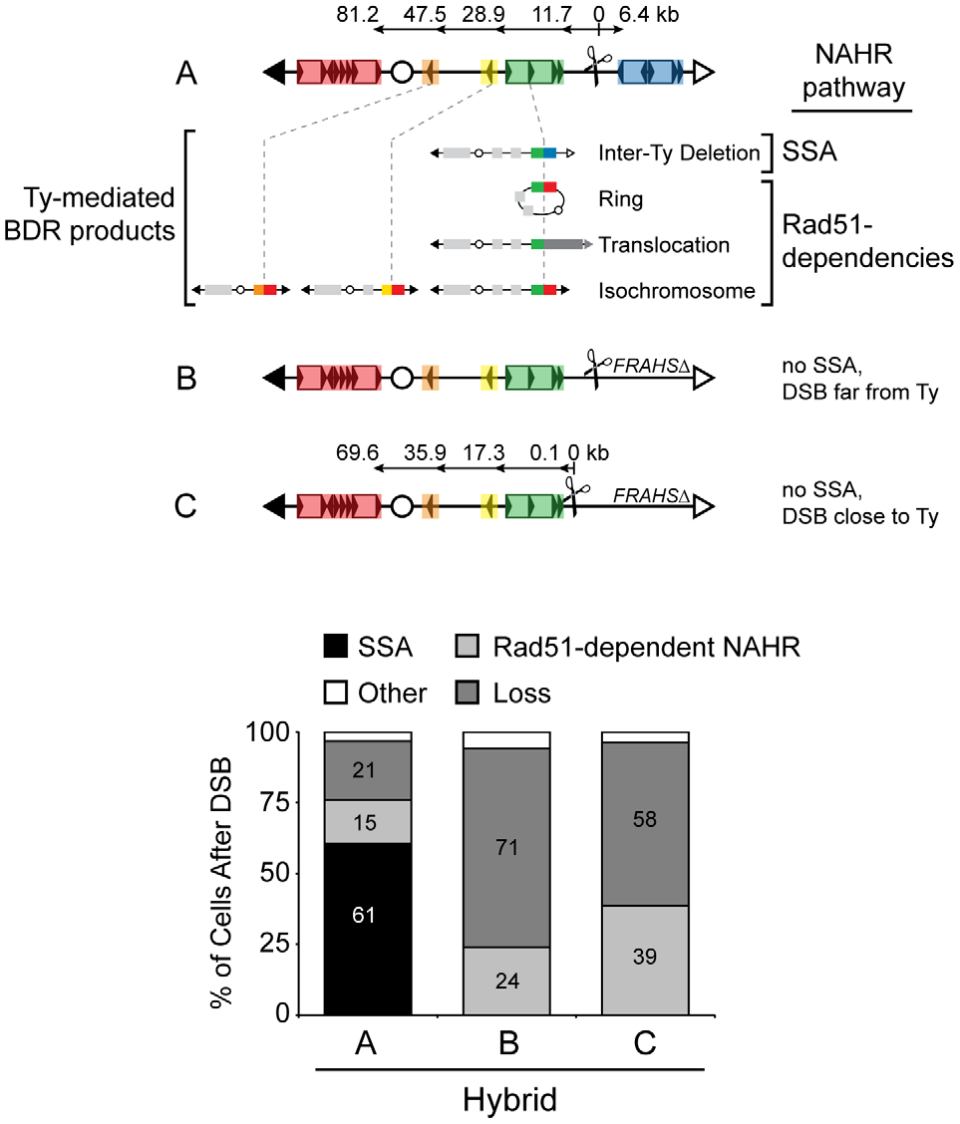
Pathway competition with DSB in unique DNA: SSA is most efficient and Rad51-dependent NAHR is inherently inefficient. Top: Schematic of three *S. cerevisiae* chromosome III configurations (A–C) analyzed in hybrid diploids. A = 163cs (MH3360), B = 163cs with *FRAHSΔ* (MH3524/MH3572/MH3573), C = 151cs with *FRAHSΔ* (MH3551). Ty-mediated BDR products for configuration A are shown below map for A. BDR recipients that mediate each rearrangement are connected with a dashed grey line to the BDR product, with intrachromosomal recipient (left of dashed grey line) and intrachromosomal donor (right of dashed grey line) partners at the recombination junctions indicated by color. Bottom: Frequencies of NAHR pathways (SSA and Rad51-dependent) and chromosome loss after a DSB in hybrid diploids strains with configuration A–C. Inter-Ty deletions are ‘SSA’; rings, translocations, and isochromosomes are ‘Rad51-dependent NAHR’; chromosome loss is ‘Loss’, and remaining outcomes (other and allelic in Table 2) are ‘Other’.

We first eliminated the dominant SSA pathway by deleting the *FRAHS* donor (*FRAHSΔ*, B in Figure 6) and looked for compensation through the remaining rearrangements. These rearrangements are grouped as Rad51-dependent NAHR since rings show full Rad51-dependency while isochromosomes and translocations have partial Rad51-dependency (Table 1). While some Rad51-dependent rearrangements show a modest increase (rings increase 3±1% to 11±3%, Table 2), the majority of cells cannot repair the DSB at 163cs without SSA, resulting in chromosome loss (71±3% loss, Figure 6). One possibility for this repair inefficiency is that the DSB is too far from the Ty recipients (at least 11.7 kb from the break site) to effectively activate the recipients in Rad51-dependent NAHR pathways. This would be consistent with evidence that Rad51 binding is limited to about 5 kb on either side of a DSB [31]. We then repositioned the I-*Sce*Ics at 151cs, within 0.1 kb of the *RAHS* recipient in the *FRAHSΔ* strain (C in Figure 6), in order to enhance Rad51 presynaptic filament assembly onto *RAHS*. Although a modest increase in Rad51-dependent rearrangements was observed, the majority of cells after the DSB at 151cs with *FRAHSΔ* cannot efficiently repair the chromosome in the absence of SSA (58±2% loss, Figure 6). These data reveal that Rad51-dependent NAHR pathways induced by a DSB in unique sequences (163cs or 151cs) are inherently inept at repairing the DSB using Ty1/Ty2 elements. Taken together, for a DSB in unique DNA, the efficiency of the SSA pathway coupled with the inefficiency of Rad51-dependent NAHR pathways generates the intrachromosomal position bias and preferential usage of Ty recipients and donors.

### Mutagenic potential of DSBs in the genome

**Figure 7:**
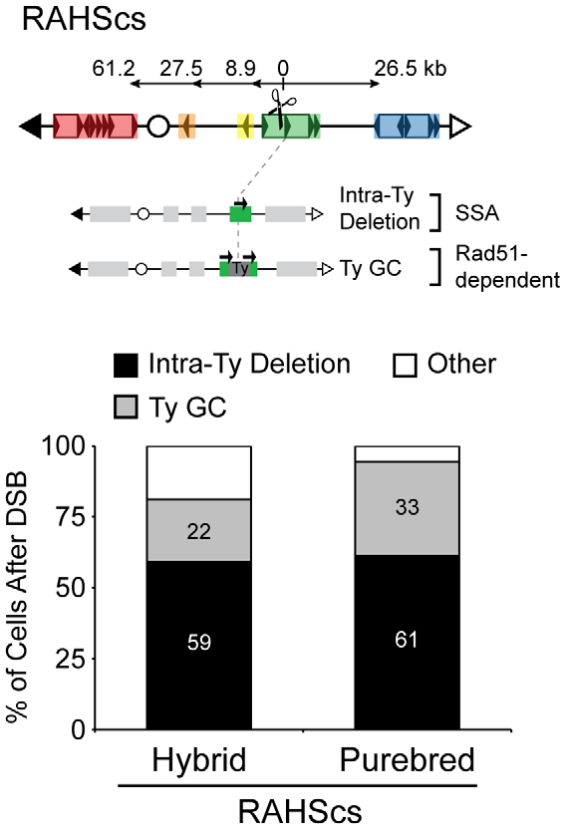
Pathway competition with a DSB in repetitive DNA: SSA and gene conversion (GC) predominate. Top: Schematic of *S. cerevisiae* chromosome III with I-*Sce*Ics inside *YCRWTy1-2* of *RAHS* (referred to as RAHScs). Two main repair products resulting from a DSB at RAHScs are shown below. (1) Intra-Ty deletion likely occurs through SSA within *RAHS*, indicated by the presence of only one black arrow at *RAHS* (referred to as ‘intra-Ty’ to distinguish from ‘inter-Ty’ deletions that occur between *RAHS* and *FRAHS*). (2) Ty GC likely occurs through a Rad51-dependent pathway and maintains *RAHS* size, indicated by two black arrows present at *RAHS* and grey Ty repair patch. Bottom: Frequencies of Intra-Ty deletion and Ty GC events after DSB at RAHScs in hybrid (MH3768) and purebred (MH3764) diploids. ‘Other’ refers to inter-Ty deletions, isochromosomes, rings, translocations, other NAHR, allelic, and loss (see Table 2).

Our findings show that the I-*Sce*I-induced DSB in unique DNA (147cs, 151cs, or 163cs) generates substantial NAHR between Ty repeats, giving rise to a broad spectrum of rearrangements through BDR in the purebred diploids. This is in contrast to current models that propose that break-proximal sequences determine the outcome, where DSBs in unique DNA lead to AHR (between sisters or homologs) and DSBs in repetitive DNA can lead to NAHR [32]. To assess the relative consequence of DSBs in unique versus repetitive DNA, we repositioned the I-*Sce*Ics into the *RAHS* locus (called RAHScs, Figure 7) and used our nonselective assay to measure all possible outcomes after the DSB at RAHScs in hybrid and purebred diploids. From the repair clones generated in our assay, we further characterized two Ty-mediated products that exclusively arise with the DSB at RAHScs, intra-Ty deletions and Ty GC. These Leu^+^Hyg^S^Ura^+^ repair clones are distinguished from each other by assaying *RAHS* locus size using PFGE/Southern analysis (Figure S7). In comparison to the wild-type *RAHS* size, we observe a smaller *RAHS* size for intra-Ty deletion events and a similar *RAHS* size (with only the removal of the small nonhomologous 1.6 kb I-*Sce*cs/*HYG* ends) for Ty GC events.

Similar to results with the DSB at 163cs, SSA dominates the NAHR pathway competition, with 66% and 61% of cells after DSB at RAHScs generating Ty-mediated deletions in hybrid and purebred diploids, respectively (Table 2). SSA again imposes a strong intrachromosomal position bias, dictating recipient and donor preferences. The internal deletions from RAHScs, however, can be generated between the *RAHS* recipient and two different Ty donors, sequences within *RAHS* itself (referred to as intra-Ty) and *FRAHS* (now referred to as inter-Ty). All of the internal deletions in purebred diploids are intra-Ty events (61±9%) whereas in hybrid diploids, 59±9% are intra-Ty and 7±5% are inter-Ty (Figure 7 and Table 2). This is consistent with previous work describing a proximity effect during SSA using model repeat donors, with break-proximal donors preferred over break-distal donors [7].

In addition to the events observed with a DSB at 163cs, we find that the second most frequent event after DSB at RAHScs is Ty GC. 22±8% and 33±10% of cells after DSB at RAHScs lead to Ty GC events in hybrid and purebred diploids, respectively (Figure 7). The lower frequency of Ty GC relative to intra-Ty deletions measured in our diploids are in agreement with those events measured using an HO-induced DSB inside Ty1 in *S. cerevisiae* haploids [33]. Ty GC occurs through the coordination of a two-ended strand invasion event into a Ty donor, which is not a possibility when the DSB initiates in unique DNA (as for 163cs). These GC events in the hybrid diploids must be mediated by a non-allelic Ty donor from the *S. cerevisiae* genome (since *S. bayanus* lacks Ty1/Ty2), which likely occurs in purebred diploids as well [16]. Thus, paradoxically, NAHR efficiently mediates conservative repair when a DSB occurs in repetitive DNA.

**Figure 8:**
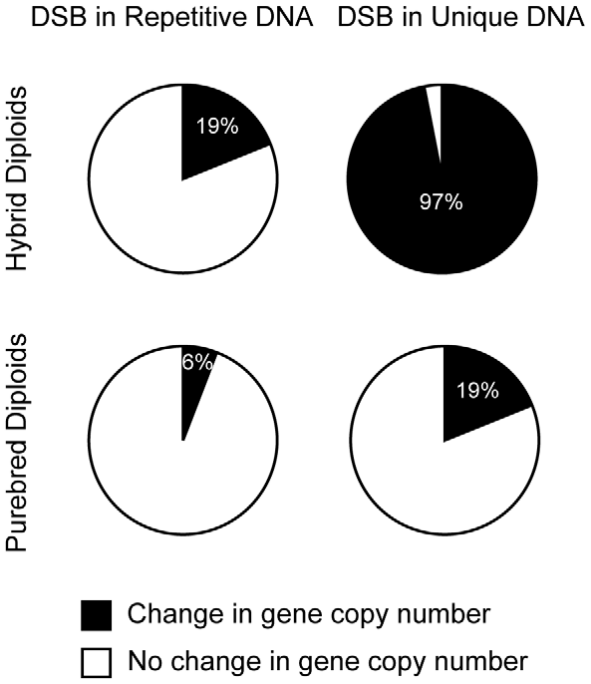
DSBs in unique DNA are more mutagenic than DSBs in repetitive DNA. Frequencies of outcomes after DSBs in repetitive DNA (at RAHScs) in hybrid (MH3768) and purebred (MH3764) diploids versus DSBs in unique DNA (at 163cs) in hybrid (MH3360) and purebred (MH3359) diploids. Outcomes are categorized into two classes: (1) ‘Change in gene copy number’ (black) are inter-Ty deletions, translocations, chromosome rings, isochromosomes, other NAHR, and chromosome loss (percentage indicated in white text), (2) ‘No change in gene copy number’ (white) are intra-Ty deletion, Ty GC, and allelic HR.

Having completed our analyses of a DSB within a Ty1 repeat, we can now compare its impact to a DSB in unique DNA on genome integrity. We categorized the outcomes of the I-*Sce*I-induced DSB at RAHScs and at 163cs into two groups: (1) change in gene copy number (inter-Ty deletion, isochromosome, ring, translocation, and chromosome loss) and (2) no change in gene copy number (intra-Ty deletion, Ty GC, and allelic). This comparison reveals that the DSB in unique DNA is 3 to 5-fold more likely to cause a change in gene copy number than the DSB in repetitive DNA (increases from 19% to 97% in hybrid diploids and 6% to 19% in purebred diploids, Figure 8). Thus, distinct from models that highlight the role of DSBs inside repeats in mediating genome rearrangements, our results suggest that the relative mutagenic potential of a DSB in the genome actually decreases when the break occurs within repetitive DNA. Furthermore, this finding suggests that DSBs in unique DNA are more likely to lead to mutagenic rearrangements than DSBs in repetitive DNA.

## Discussion

We report a novel genome-wide system in budding yeast to study non-allelic homologous recombination (NAHR) between natural repeats. While previous assays isolate aspects of competitive repair addressed here, our system gauges the competition between all parameters concurrently, as what naturally transpires in a cell. The value of this new approach is evidenced by the surprising features of NAHR our system reveals. Remarkably, in purebred diploids, DSBs within a long stretch of unique sequences are not always repaired by allelic homologous recombination (AHR) as previously assumed. Rather, 17% of these DSBs repair by NAHR. This NAHR arises because the DSB activates Ty recipients 12 to 48 kb distal from the break site to recombine with non-allelic Ty donor sequences. Robust NAHR through break-distal recombination (BDR) is supported by a previous study of bridge-breakage-fusion in diploid budding yeast by Malkova and colleagues [34].

In this and the previous study, competition between BDR-dependent NAHR and AHR occurs after an endonuclease-induced DSB. In diploids, endonucleases can cleave one homolog prior to DNA replication and both its sister chromatids after DNA replication, thereby eliminating the sister chromatid as a donor for AHR. Therefore, the only AHR donor is the uncut homolog. However, a homolog is also the only AHR donor for repair of spontaneous DSBs that occur on unreplicated DNA in G1 or S. Indeed, recent evidence suggests that spontaneous DSBs occur on unreplicated DNA [35]. We suggest that spontaneous DSBs in unique unreplicated DNA are also likely to induce robust BDR-dependent NAHR.

The fact that break-distal Ty sequences undergoes frequent NAHR reveals two surprising features of recombination that have important mechanistic implications for current models of recipient activation and choice. The first surprise is that distal Ty repeats are activated as recipients at all (presumably by becoming single-stranded) when break-proximal ssDNA can undergo AHR. Indeed, a recent study in diploid yeast suggests that ssDNA is generated at least 10 kb from a DSB before its repair is complete [36]. To explain this extensive break-distal resection, we suggest that a step after resection must be slow, such as the homology search for donor sequences. A slow homology search would provide time for break-distal sequences to be resected and compete with previously resected break-proximal sequences. Such a slow homology search is consistent with studies suggesting the slow diffusion of chromosomal sequences [37].

The second surprise is the disproportionate use of very small break-distal Ty sequences as recipients for NAHR. They would represent only a very small proportion of the entire block of resected DNA, which can all act as a recipient for AHR. We suggest that the smaller Ty recipients encounter their potential Ty donors first because chromosome territories [38] generate a high local concentration of potential intrachromosomal Ty donors. In contrast, the larger allelic recipients must travel further to partner with allelic donors on the homolog. Consistent with this model, almost all NAHR rearrangements through break-distal Ty recipients result from pairing with intrachromosomal Ty donors.

Along with recipient usage, our genome-wide system reveals the role sequence homology and genomic position play in NAHR donor choice. We find that the Ty donors chosen by a recipient are not among the most homologous in the genome by the criteria of either percent identity or longest block of uninterrupted identity. Rather the primary determinant of NAHR donor choice is local proximity. We observe a ∼50-fold preference for Ty repeat donors on the same chromosome over different chromosomes. This intrachromosomal NAHR preference is consistent with previous studies [16]–[19], although the magnitude of this preference differs, possibly due to specific configurations of repeats relative to a break site, as observed in our studies. However, in contrast to previous work, our study shows this intrachromosomal bias occurs under conditions that allow unrestricted choice of repair pathways and partners amongst a natural repetitive family. Interestingly, Ty1/Ty2 elements are preferentially inserted within 750 bp upstream of tRNA genes [39], and dispersed tRNA genes cluster together [40]. Our results suggest that possible Ty interchromosomal contacts mediated by tRNA clustering is not sufficient to overcome an intrachromosomal bias. It will be interesting to see whether higher-order chromosome organization may influence donor repair choice of natural repeats when only interchromosomal donors are available for NAHR.

Our system also provides insights into the preferred repair pathways that act on a family of natural repeats. We show that NAHR occurs mostly by the SSA pathway whether DSBs occur in unique sequences or a Ty repeat. The robustness of SSA is consistent with previous studies using model repeats [18], [23], [30], [41], [42]. Since repair of a single DSB by SSA will occur through an intrachromosomal donor, the predominance of SSA helps explain the preferential usage of intrachromosomal donors and the resulting preference for intrachromosomal NAHR.

Importantly, our pathway analysis of NAHR also helps explain one of the most surprising and striking observations of this study: DSBs that occur *outside* repeat clusters are more mutagenic than DSBs that occur *inside* repeat clusters. This seemingly counterintuitive observation arises because DSBs that occur inside a Ty have better options for repair, both in efficiency of pathways and favorably positioned donors. DSBs within the Ty predominately repair through two highly efficient pathways, SSA within the Ty locus or GC with preferred intrachromosomal Ty donors [16]. These types of repair preserve gene copy number since neighboring unique genes are unaffected. Since SSA and GC are compensatory pathways [22], it is possible that DSBs inside repetitive elements that cannot undergo SSA (i.e. solo insertion of LINE-1) efficiently repair through GC events [43]. A recombination execution checkpoint has been suggested to maintain genome integrity by ensuring the coordination of two-ended strand invasion events during GC for conservative repair [28]. Consistent with this, our results suggest that NAHR through GC between natural repeats is a major mechanism that limits changes in genome structure.

In contrast, DSBs in unique sequences that repair predominately through GC with the homolog is not as effective in limiting detrimental rearrangements. As the search for the interchromosomal homolog allows for more time to activate a break-distal Ty as a recipient, BDR occurs more frequently through SSA between distinct Ty loci or one-ended events through the BIR pathway. In this situation, SSA always, and BIR often times, change the copy number of neighboring unique genes. Hence, this opens up the possibility that DSBs in unique sequences, rather than repeats, may generate spontaneous or irradiation-induced NAHR-dependent rearrangements observed in yeast [32], [44]. Similarly, NAHR-dependent rearrangements in the human genome may also occur by a DSB in the surrounding unique DNA followed by BDR-dependent NAHR. If so, then the recombinant junction would not coincide with the site of the initiating lesion. Therefore, analysis of NAHR junctions alone may miss underlying mechanisms for genome rearrangements. Examining broad regions around NAHR junctions could potentially identify fragile sites that predispose a locus to recurrent instability, contributing to genetic diversity and disease.

## Materials and Methods

### Yeast strains

Standard yeast genetic and molecular biology methods were used [45]. All *S. cerevisiae* strains were derived from BY4700 (*MATa ura3Δ0*), BY4716 (*MAT*α *lys2Δ0*), or BY4704 (*MATa ade2Δ::hisG his3Δ200 leu2Δ0 lys2Δ0 met15Δ0 trp1Δ63*) [46]. All *S. bayanus* strains were derived from a *S. bayanus* prototroph received as a gift from Ed Louis. Deletion of the *HO* gene and auxotrophic markers were introduced by transformation to generate a number of haploid *S. bayanus* strains for laboratory use, including MH3399 (*MATa hoΔ::hisG ura3Δ::NAT leu2Δ::NAT ade2Δ::hisG*), YZB9-4B1 (*MATa hoΔ::KAN ura3Δ::NAT leu2Δ::NAT*), YZB5-102 (*MAT*α *hoΔ::KAN lys2-1*) (this study, [47]). Since *S. bayanus* is sensitive to high temperatures, the following modifications were made to the high efficiency yeast transformation protocol [48] for *S. bayanus* and hybrid diploids strains: room temperature incubation of transformation mix for 30 minutes, 5 minute heat shock at 42°C, and 5 minute rest at room temperature following heat shock.

Except for some noted below, insertion/knockout constructs were generated through one-step transformation of a PCR amplified linear construct. Each primer for these constructs included ∼50 bp of homology to target for genomic integration and ∼20 bp that anneal to a plasmid template for the amplification of a selectable marker [pAG32-hphMX4 (Hygromycin B), pAG25-ClonatMX4 (Clonat), pFA6a-kanMX4 (Kanamycin), or pMPY-ZAP (*hisG-URA3-hisG* pop-in/pop-out construct)]. One primer of each of the I-*Sce*I cut site primer pairs also included the 30 bp I-*Sce*I recognition sequence from [49]. For RAHScs, the primers included linkers to amplify an AgeI-I-*Sce*Ics/*HYG*-ClaI fragment, which was digested and ligated into AgeI-ClaI site of pFT1 (derived from p150Ty, this study). The resulting plasmid, called pFT1-SceIcs, was double-digested with NotI and KpnI and a 10.2 kb purified NotI-KpnI fragment was used for transformation to create RAHScs. For *FRAHSΔ::hisG*, three primer pairs (FRAHSΔ-left, FRAHSΔ-middle, FRAHSΔ-right) were used to generate three overlapping fragments that were co-transformed. Sequences for gene knockout primers are available upon request. All other strain construction primers included in Table S2. All genome manipulations were performed in haploid strains, and all constructs were verified by Southern blot analysis. Pairs of *S. cerevisiae* and *S. bayanus* haploids were mated to generate the desired purebred and hybrid diploids, and then transformed with the I-*Sce*I expression plasmid (see below). All experiments in this study were performed at 23°C unless noted otherwise.

### Media and reagents

Yeast strains were grown in YEP, SC-ADE, SC-ADE-URA media supplemented with 2% dextrose (D), 2% lactic acid 3% glycerol (LAG), 0.3 mg/ml Hygromycin B (HYG), as indicated. YEPD media was supplemented with 10 µg/ml adenine. Glucose and glycerol was purchased from EMD Biosciences, lactic acid (40% v/v stock, [pH 5.7]) from Fisher Scientific, and Hygromycin B (HYG) from Roche. SC dropout powders were homemade from amino acids purchased from Sigma-Aldrich.

### I-*Sce*I expression plasmids

The GALp-I-*Sce*I construct was from pWJ1320 [49], a gift from Rodney Rothstein. pMH5 was derived from pWJ1320 (2 micron-based) by deleting a 2.0 kb EcoO109I fragment containing *URA3* marker. pMH6 (2 micron-based) and pMH7 (CEN-based) were created by ligating the 2.0 kb SalI fragment from pWJ1320 (containing the GALp-I-*Sce*I expression construct) into the unique SalI site of pRS422 and pRS412, respectively. pMH6 and pMH7 were generated to include a larger promoter sequence for the *ADE2* marker, however, all plasmids yielded similar results.

### Induction of I-*Sce*I site-specific DSB

A single colony from SC-ADE-URA+D+HYG plates [to select for GALp-I-*Sce*I expression plasmid (Ade^+^) and no DSB (Hyg^R^Ura^+^)] was used to inoculate SC-ADE-URA+D for a 5 ml starter culture that was grown to saturation. A small volume of the starter was used to inoculate SC-ADE+LAG cultures and these cultures were grown for more than two doubling to exponential phase [OD(600) ∼1.0]. For the uninduced control, immediately before DSB induction, an aliquot was appropriately diluted in water and plated onto YEPD for individual colonies (uninduced frequencies are subtracted out of induced frequencies, see below). To induce the DSB, galactose (20% v/v stock) was added to a final of 2% and after two hours, the cultures were diluted in water and plated onto YEPD for individual colonies (referred to as clones). Plates were incubated at 23°C for 3–5 days.

### Determine frequencies of chromosome III repair or loss after an I-*Sce*I–induced DSB

YEPD platings from uninduced and induced were first replica plated onto YEPD or 2% agar plates. This replica plate was then immediately used on a fresh velvet to replica onto YEPD+HYG, SC-URA+D, and SC-LEU+D plates. These marker plates were incubated at 23°C for 2–4 days. Each colony from the original YEPD plate was scored for the presence or absence of chromosome III markers (*LEU2*, *HYG*, *URA3*) by growth or no growth on marker plates. Assessment of the heterozygous markers (present on the *S. cerevisiae* homolog with the I-*Sce*Ics) determines whether the founding cell had experienced an I-*Sce*I-induced DSB (leading to the Hyg^S^ phenotype) followed by chromosome repair [Hyg^S^ and Leu^+^Ura^+^ (class I) or Hyg^S^ and Leu^+^Ura^−^ (class II)] or chromosome loss [Hyg^S^ and Leu^−^Ura^−^ (class III)]. The Hyg^S^ phenotype most likely occurs through the removal of the nonhomologous ends (1.6 kb I-*Sce*Ics/*HYG* construct), which is a natural and efficient step during HR repair [50], [51].

The following three steps were used to calculate frequencies of repair and loss events. First, the numbers of clones that fell into each genetic class (I, II, III) out of the total number of clones scored were calculated as percentages for both uninduced and induced cultures. Second, uninduced percentages were subtracted from induced percentages to eliminate events that occurred before galactose addition. Occasionally, cultures with high background frequencies (>50% of clones were Hyg^S^ in uninduced cultures) were observed and not used. Hyg^S^ phenotypes before galactose induction are due to leakiness of the galactose promoter during nonrepressive growth (see Figure S3). Third, the total percentage (class I + class II + class III) was normalized to 100%. A third potential repair class, Hyg^S^ and Leu^−^Ura^+^, arose so infrequently (<1% in wild-type purebred and hybrid diploids) that it was omitted from these calculations.

### Determining type of repair after an I-*Sce*I–induced DSB

Single repair clones (class I and II) from SC-LEU+D marker plates were restruck for individual isolates onto fresh SC-LEU+D plates to ensure clonality (i.e. possible mixing during replica plating process). One isolate from this restreak was used to inoculated YEPD media and grown to saturation for the subsequent isolation of genomic DNA for PFGE/Southern analysis using a *LEU2* probe (see below). Hybridization that resulted in wild-type chromosome III size (purebred diploids at 341 kb, hybrid diploids at 320 kb) was identified as AHR and those with an altered chromosome III size, indicative of a rearrangement, were classified as potential NAHR. The structures of the chromosome III rearrangement structures were first determined in wild-type hybrid diploids (MH3360) due to the advantage of no signal from an uncut homolog.

#### Internal deletions

Rearrangements in genetic class I from MH3360 were determined to be internal deletions mediated by *RAHS* and *FRAHS* and based on three pieces of evidence: 1) 18 repair clones analyzed by PFGE/Southern, which indicated a ∼20–30 kb decrease in *S. cerevisiae* chromosome III size compared wild-type (341 kb) as would be predicted for an internal deletion between *RAHS* and *FRAHS*, 2) Same 18 repair clones were subjected to PCR analysis using *S. cerevisiae* specific primers that flank *RAHS* (RAHS-L and RAHS-R) and *FRAHS* (FRAHS-L and FRAHS-R), which resulted in PCR products at the two outer sides of *RAHS* and *FRAHS* and no PCR product at the inner two sides (whereas all bands appear in the wild-type control) (primer sequences in Table S2). At least one hybrid and purebred internal deletion clone was further analyzed by long-range GeneAmp XL-PCR (Applied Biosystems) with primers that amplified the predicted *RAHS*-*FRAHS* deletion junction (primer sequences in Table S2) and 3) *FRAHSΔ* in MH3524/MH3572/MH3573 (eliminates donor) nearly abolished genetic repair class I (<4% of cells after DSB).

#### Isochromosomes, chromosome rings, and translocation

Rearrangements in genetic class II were determined to be mainly composed of three structures. 52 repair clones in class II from MH3360 were classified into three groups based on PFGE/Southern hybridization pattern: Group W for hybridization in well, Group L for larger (>340 kb), and Group S for smaller (210–280 kb). The recipient Ty loci used to mediate the rearrangements were localized to the recombinant junction by PCR analysis on 21 repair class II clones from MH3360 using primer pairs that flank *YCRCdelta6* (YCRCdelta6-L and YCRCdelta6-R), *YCRCdelta7* (YCRCdelta7-L and YCRCdelta7-R), and *RAHS* (RAHS-L and RAHS-R) (primers sequences in Table S2). Group W was further determined to be chromosome rings mediated by *RAHS* and *LAHS* based on the following observations: (1) Leu^+^ phenotype, yet PFGE/Southern analysis indicated no *LEU2* probe hybridization in the lane, but strong hybridization in well, (2) Unlike control samples, Southern analysis on four clones from MH3346 (same as MH3360, but I-*Sce*Ics/HYG construct is inverted) showed an absence of signal from probes that hybridize to restriction fragments near telomere ends, (3) Digestion of four PFG agarose plugs with PacI from MH3346 followed by PFGE/Southern analysis resulted in the release of an ∼80 kb fragment that hybridizes to *LEU2* probe concomitant with loss of hybridization signal to the well, (4) aCGH on one clone generated from MH3346 showed sequence loss of all left and right telomere-proximal sequences adjacent to *LAHS* and *RAHS*, (5) MH3398 (*LAHS*Δ, eliminates ring donor) and MH3471 (147cs, eliminates ring recipient) abolished Group W by PFGE/Southern analysis, and (6) at least one hybrid and purebred ring clone was further analyzed by long-range GeneAmp XL-PCR using primers that amplified the predicted *RAHS*-*LAHS* ring junction (primer sequences in Table S2). For Group L, PFGE/Southern analysis was repeated on 12 clones from strain MH3360 and MH3398 (*LAHS*Δ enriches for translocations in class II) under conditions that separated all *S. cerevisiae* chromosomes. Majority of clones (9 out of 12) were ∼485 kb and aCGH on two of these clones suggested a translocation mediated between *RAHS* and *YJRWTy1-1/YJRWTy1-2* locus from chromosome X. For Group S, PCR analysis localized to the recombinant junction three different Ty recipient loci, *YCRCdelta6*, *YCRCdelta7*, and *RAHS* corresponding to Group S size subclasses of 210–230 kb, 240–255 kb, and 260–280 kb, respectively. At least one hybrid and purebred isochromosome clone was further analyzed by long-range GeneAmp XL-PCR using primers that amplified the predicted *YCRCdelta6-LAHS* and *YCRCdelta7-LAHS* junction (primer sequences in Table S2). Group S were further determined to be isochromosomes based on (1) aCGH on one clone from MH3346 indicated a 2-fold increase to of left arm adjacent to *LAHS* and loss of all sequences to right of *YCRCdelta6*. (2) MH3398 (*LAHSΔ*, eliminates isochromosome donor) abolished Group S by PFGE/Southern analysis. (3) MH3471 (147cs, eliminates *RAHS* recipient) abolishes 260–280 kb-sized clones (*RAHS*-mediated isochromosomes) by PFGE/Southern analysis.

These aCGH and PCR analyses of chromosome III rearrangements revealed that many specific rearrangements reoccur and have signature mobility on PFGs. Representative clones were subjected to aCGH and PCR analyses to validate the use of signature mobilities as a diagnostic tool for rearrangements. These signature mobilities matched the mobilities of the rearranged chromosome III from repair clones found in the mutant hybrids as well as wild-type and mutant purebreds. Therefore in these other diploids, we could use the mobility of the rearrangement to identify the type of rearrangement as well as the specific recipient and donor loci.

### Calculation of frequencies of outcomes after an I-*Sce*I–induced DSB

Frequencies were calculated in three steps. 1) Frequencies of genetic classes (I, II, III) of uninduced cultures were subtracted from frequencies of induced cultures to eliminate events that occurred prior to galactose addition (described in more detail above, frequency of chromosome loss determined here). 2) For the repair events, the fraction of each type of repair (i.e. allelic, internal deletion, etc) among the total PFG plugs analyzed from its corresponding genetic class (I or II) was calculated. 3) For the repair events, the genetic class frequency (step one) was multiplied by the fraction of each repair type in that genetic class (step two). For example, in wild-type purebred diploids (MH3359), 85.7% of Hyg^S^ clones (n = 1062) were class I (Leu^+^Hyg^S^Ura^+^). 5 out of 32 random repair clones of class I were classified as internal deletions by PFGE/Southern analysis, so the frequency of internal deletions in MH3359 is 5/32(85.7%) = 13.4%.

### PFGE/southern analysis

Yeast genomic DNA was prepared in 1% low-melting agarose plugs (SeaPlaque 50100) as previously described [52] and resolved on 1% agarose gel (Bio-Rad 162-0138) in 0.5XTBE using Bio-Rad CHEF-DR III System. To optimize resolution between *S. cerevisiae* and *S. bayanus* chromosome III the following parameters were used: 6 V/cm, 120° angle, 1–25 s switch times, 24 hours at 14°C. To assess yeast whole genome karyotypes (i.e. for translocations), the parameters were the same except for 60–120 s switch times. Gels were blotted using GeneScreen Plus membrane (Perkin Elmer NEF988) and probed with a 1.3 kb fragment from the *S. cerevisiae LEU2* locus amplified using the U2-FOR/U2-REV primer pair (Table S2).

### Calculation of standard error of the mean (SEM)

To calculate SEMs for the repair outcomes, the following numbers were used: (a) average frequency of Leu^+^Hyg^S^Ura^+^ genetic class I, (b) average frequency of Leu^+^Hyg^S^Ura^−^ genetic class II, (c) total number of Leu^+^Hyg^S^Ura^+^ (class I) plugs analyzed by PFGE/Southern analysis, (d) total number of Leu^+^Hyg^S^Ura^−^ (class II) plugs analyzed using PFGE/Southern analysis, (e) number of Leu^+^Hyg^S^Ura^+^ (class I) plugs of a particular repair outcome (i.e. allelic, internal deletion), (f) number of Leu^+^Hyg^S^Ura^−^ (class II) plugs of a particular outcome (i.e. ring, translocation, isochromosome). SEM was calculated in two steps. First, the initial SEM was calculated using the formula SQRT(pq/n), where p =  fraction of a particular repair outcome observed by PFGE/Southern analysis over total analyzed from that class (e or f divided by c or d, respectively), q = 1-p, and n =  total number of repair clones analyzed by PFGE/Southern analysis from that corresponding class (c or d). Second, the final SEM was calculated by weighting the SEM with the corresponding genetic class frequency (initial SEM multiplied by a or b).

The rationale for this method was to be most stringent by using the smallest n (d or e). In the following cases e or f was assigned the number 1: (1) when all Leu^+^Hyg^S^Ura^+^ plugs were deletions (i.e. in hybrid diploids), (2) no products appear in any plugs analyzed (i.e. rings in *rad51Δ/rad51Δ* mutant), (3) genetic class is 0 (i.e. Leu^+^Hyg^S^Ura^−^ class II in *rad52Δ/rad52Δ* hybrid diploids), (4) when no plugs analyzed (i.e. Leu^+^Hyg^S^Ura^−^ class II in *rad52Δ/rad52Δ* purebred diploids). For case 1, the error was estimated by assuming the next plug would not be that particular outcome. For case 2, 3, and 4, the upper bound was estimated by assuming the next plug would be that particular outcome. In the case where repair outcomes came from both the Leu^+^Hyg^S^Ura^+^ and Leu^+^Hyg^S^Ura^−^ genetic classes (i.e. other, allelic in purebred diploids), “final SEMs” were calculated as described above and then “final SEMs” from each class was added together for the reported SEM. To calculate SEMs for chromosome loss, the formula SD/SQRT(n) was used where SD (standard deviation) =  SD of the frequency of Leu^−^Hyg^S^Ura^−^ clones from different isolates and/or DSB-inductions (same experiment used to generate numbers for a and b above) and n =  total number of different DSB-inductions performed for that particular strain (ranging between 2 to 8).

### Viability

Exponential cultures in –ade +2% lactic acid +3% glycerol were appropriately diluted in water and the same volume was plated on –ade +2% galactose and –ade +2% glucose. Plates were incubated at 23°C. Percent viability was calculated as the number of colony forming units on galactose divided by the number of colony forming units on glucose.

### Array CGH

aCGH methods were performed as previously described [53]. *S. cerevisiae*/*S. bayanus* hybrid microarrays were custom designed and printed by Lewis-Sigler Institute Microarray Facility at Princeton University.

### Sequencing of LAHS, RAHS, FRAHS from S288C background

Numerous studies have brought to light unannotated Ty elements on chromosome III [34], [44], [54]–[56], with a few studies publishing a limited restriction digest map of the Ty structure in these regions [44], [54], [55]. These unannotated Ty clusters were sequenced here. Each cluster was cloned from strain MH3303 (*MATa lys2Δ0 ura3Δ0*, derived from BY4716 [46]) by gap repair to create p85Ty, p150Ty, and p169Ty (see Figure S1). Each plasmid was subjected to transposon bombing using the Finnzymes Template Generation System (TGS). For each plasmid, 192 clones with different random transposon insertions were picked and sequenced with a pair of primers located at the edges of the TGS transposon to produce pairs of oppositely directed reads. 384 attempted reads were performed per yeast clone. Sequence data were processed, assembled and edited using the Phred/Phrap/Consed suite of programs [57]. Each assembly was reviewed and edited to ensure there were no discrepancies due to misplaced reads or low quality regions. The automated assembler resulted in collapses of repeats, and these were manually resolved. 16.8 kb of sequence at *LAHS*, 14.5 kb at *RAHS*, and 14.7 kb at *FRAHS* were deposited into GenBank with accession number GU224294, GU220389, and GU220390, respectively. The sequence included five additional full length Ty1s and a solo LTR, complementing the *LAHS* reference sequence in SGD and almost entirely replacing the *RAHS* and *FRAHS* reference sequence. The new sequence changes chromosome III size from 316,617 bp (in SGD) to 341,823 bp.

### Sequence comparisons of Ty1 and Ty2 elements

Sequences for all previously described Ty1, Ty2 and LTRs (delta) elements were obtained from the SGD “Non-ORF dataset” (http://downloads.yeastgenome.org/, timestamp January 5, 2010). Several corrections were made based on our resequencing and analysis: (1) addition of five Ty1 elements on chrIII (Ty1–1 through Ty1–5) (2) addition of nine delta elements on chrIII (delta16 through delta24) (3) removal of three delta elements on chrIII (*YCRWdelta8*, *YCRWdelta9*, and *YCRWdelta10*) (4) addition of one unannotated Ty1 element on chrXII (encompassing *YLR035C-A*) (5) addition of two unannotated delta elements on chrIV (LTRs for *YDRCTy1-2*).

The “Overall Identity (%)” between two sequences was determined by creating a global sequence alignment using the Needleman-Wunsch algorithm (gapopen = 10, gapextend = 0.5) as implemented in needleall v6.2.0 [58].

The “Longest Block of 100% Identity (nt)” was determined by first creating a local sequence alignment using the NCBI BLAST algorithm (match = 1, mismatch = −3, gapopen = −1, gapextend = −1) as implemented in bl2seq v2.2.18 [59]. Custom Perl scripts using BioPerl v1.6.1 iterated through each set of hits to identify the longest contiguous block of matching nucleotides [60].

Finally, the contribution of sequence similarity to donor usage is likely more complex than either overall identity or longest block of perfect identity. We therefore calculated bit scores using the BLAST heuristic, which attempts to balance length and perfect identity when searching for a shared region between two sequences that has the “most” similarity. This “Local Identity (bitscore)” was determined using blastall.

Source code and data files can be found at: http://dl.getdropbox.com/u/547386/code.zip

## Supporting Information

Figure S1 Sequencing of unannotated Ty elements at three Ty clusters on *S. cerevisiae* chromosome III. (A) Schematic of *S. cerevisiae* chromosome III showing the Ty configuration of left arm transposition hotspot (*LAHS*) [Warmington et al 1986], right arm transposition hotspot (*RAHS*) [Warmington et al 1987], far right arm transposition hotspot (*FRAHS*) [54] in a standard S288C background. These three loci are herein referred to by their original names in the literature. Unannotated Ty features are given systematic names (bold) in this study according to yeast nomenclature. Full length Tys are shown as open rectangles with triangles (LTRs) inside. Two annotated solo LTRs, *YCRCdelta6* and *YCRCdelta7*, are located between centromere (white circle) and *RAHS*. (B) Left: Images taken from SGD Gbrowser showing annotated features at *LAHS* (coordinates 81179–92378), *RAHS* (coordinates 146628–152734), and *FRAHS* (coordinates 167399–170909). The reference sequence of chromosome III was based on a composite of four different nonstandard backgrounds [Oliver et al]. Right panel: Yeast clones generated from gap repair of *LAHS*, *RAHS*, *FRAHS* in a standard S288C strain derived from BY4716 [46]. 0.8–1 kb fragments corresponding to the left (black box) and right (white box) of each Ty cluster provided the homology for gap repair. 16,785 bp at *LAHS*, 14,549 bp at *RAHS*, and 14,683 bp at *FRAHS* (pRS316 vector sequence omitted) were deposited into GenBank with accession number GU224294, GU220389, and GU220390, respectively. The deposited sequences include five full length Ty1s and a solo LTR that have not previously been included in any genome-wide Ty sequence analyses. [Warmington JR, Anwar R, Newlon CS, Waring RB, Davies RW, et al. (1986) A ‘hot-spot’ for Ty transposition on the left arm of yeast chromosome III. Nucleic Acids Res 14: 3475–3485.][Warmington JR, Green RP, Newlon CS, Oliver SG (1987) Polymorphisms on the right arm of yeast chromosome III associated with Ty transposition and recombination events. Nucleic Acids Res 15: 8963–8982.] [Oliver SG, van der Aart QJ, Agostoni-Carbone ML, Aigle M, Alberghina L, et al. (1992) The complete DNA sequence of yeast chromosome III. Nature 357: 38–46.]
(1.07 MB TIF)

Figure S2 *S. cerevisiae*/*S. bayanus* hybrid diploids are competent in DNA maintenance and repair. (A) Doubling time of yeast diploids in YEPD at indicated temperatures. Not determined (n.d.) for *S. bayanus* purebred diploids at 37°C due to temperature-sensitivity. Error bars indicate SD (n = 3). (B) Frequencies of spontaneous *S. cerevisiae* chromosome III loss in *S. cerevisiae* purebred (CC5) and *S. cerevisiae*/*S. bayanus* hybrid (BC11). Chromosome III stability genetically monitored by spontaneous loss of both *LEU2* (endogenous locus) and *URA3* integrated into *YCR025C* (same disruption used for I-*Sce*I/*HYG* construct at 163cs). Fresh 23°C overnight YEPD cultures were diluted and plated on 5-FOA, -leu+5-FOA, and YEPD to measure CFU/mL. Plates incubated at 23°C. Loss calculated as [(CFU/mL on 5-FOA) − (CFU/mL on –leu+5-FOA)] / (CFU/mL on YEPD). Error bars indicate SD. At least eight independent cultures assayed for each strain. (C) DNA damage drug sensitivity assayed by a five-fold serial dilutions. Plates incubated for 4 days at 23°C. MMS, methyl methanesulfonate.
(1.74 MB TIF)

Figure S3 Induction of I-*Sce*I endonuclease leads to Hygromycin-sensitivity. Hygromycin phenotype of clones before (−) and after (+) galactose induction in strains MH3360 and MH3359 (with GALp:I-SceI plasmid construct), and vector only control strain MH3802 (without GALp:I-SceI). Note that the majority of clones are Hyg^R^ (or no DSB) before galactose addition. The Hyg^S^ clones observed before induction may be due to leakiness of the galactose promoter during nonrepressive growth. After galactose induction, the small fraction of clones that remain Hyg^R^ (<10%) may be due to repair through nonhomologous end-joining, inefficient cutting before glucose repression, or loss of the I-*Sce*I expression plasmid. Total number of clones scored before and after galactose induction, respectively, is n = 779 and n = 999 for MH3360, n = 812 and n = 1068 for MH3359, and n = 197 and n = 349 for MH3802. Error bars indicate SD. At least two independent experiments assayed for each strain.
(0.65 MB TIF)

Figure S4 Ty elements mediate rearrangements. (A) Examples of PFG imaged by Ethidium Bromide staining and Southern blotting using *LEU2* probe in repair clones from hybrid diploids (MH3360) with or without a DSB. Noted are the size markers (lambda, internal chromosomes) used to determine approximate sizes of bands. Noted below gels is the approximate repair size class. Sizes on PFGE/Southern correlate with rearrangement type and were used to assign rearrangements in hybrids and purebreds diploids. (B) Chart summarizing examples of PCR analysis to determine presence of chromosome III sequences in hybrid repair clones shown in (A). *S. cerevisiae* chromosome III primer pairs from CENIII to *FRAHS* identify break-distal Ty recipient locus. For example, in R87 the sequence left of *YCRCdelta6* was present (black box) but right of *YCRCdelta6* was absent (marked with X), indicating that *YCRCdelta6* was at the recombination junction. (C) Release of chromosome rings (R51 and R53) from PFG well by PacI digestion in repair clones generated by hybrid MH3346. Note that strain MH3346 contains an inverted I-*Sce*Ics/HYG construct, but behaves like MH3360. Southern blot using *LEU2* probe to PFG with untreated plug samples (four left lanes) and PacI digested plug samples (four right lanes). In untreated R51 and R53, *LEU2* probe hybridized to the well with no discrete hybridization in the lane. PacI treated R51 and R53 showed hybridization of a discrete band in the lane. R60 (isochromosome mediated by *YCRCdelta7)* and R63 (allelic) are also shown for comparison. (D) Examples of aCGH karyoscopes of repair clones from hybrid diploids (MH3346). From the whole genome, only *S. cerevisiae* chromosome III and relevant chromosomes are shown along with the corresponding *S. bayanus* homeolog. (E) Examples of the PCR analysis using primers that flank the predicted recombinant junction for the Ty-mediated rearrangements. Bands were amplified using long-range PCR across the junctions for at least one hybrid (H) and one purebred (P) repair clone representing each major intrachromosomal rearrangement class (internal deletion, ring, isochromosome). Genomic DNA from purebred diploids (MH3357) was used as a negative PCR control. A background band is observed for deletions in the MH3357 control, which may be real or due to PCR template switching.
(1.95 MB TIF)

Figure S5 Presence of near perfect identity at the DSB does not prevent break-distal recombination. (A) Map of chromosome III homologs in I-*Sce*Ics/I-*Sce*Ics-mut purebreds (MH3525). *MATa* homolog contains the same 1.6 kb HYG/I-*Sce*Ics construct at the allelic position of the 163cs, except for a G to A base pair mutation in the I-*Sce*I cut site (mutant 320 in [Monteilhet et al]) that abolishes I-*Sce*I recognition (called I-*Sce*Ics-mut). (B) PFGE/Southern blot using *LEU2* probe (hybridizes to both homologs) on Leu^+^Ura^+^ and Leu^+^Ura^−^ random clones after galactose induction. Break-distal recombination using *YCRCdelta6* (*∂6*), *YCRCdelta7* (*∂7*), and *RAHS* results in Ty-mediated rearrangements, indicated by the repair size class. (C) Frequencies of Ty-mediated rearrangements after galactose induction in purebred MH3525. Note that *HYG* marker cannot be scored therefore calculated frequencies are likely an underestimate due to a background of uncut cells. For reference, 9% of cells remain uncut (Hyg^R^) after galactose induction in wild-type purebred strain MH3359 (see Figure S3). 2116 clones after galactose induction were phenotyped. PFGE/Southern analysis was further performed on 24 Leu^+^Ura^+^ and 23 Leu^+^Ura^−^ random clones (shown in B). Error bars indicate SEM. [Monteilhet C, Perrin A, Thierry A, Colleaux L, Dujon B (1990) Purification and characterization of the in vitro activity of I-Sce I, a novel and highly specific endonuclease encoded by a group I intron. Nucleic Acids Res 18: 1407–1413.]
(1.71 MB TIF)

Figure S6 Break-distal recombination (BDR) occurs with an I-*Sce*I-induced DSB on *S. cerevisiae* chromosome V. (A) Map of *S. cerevisiae* chromosome V indicating I-*Sce*I cut site (cs) with *HYG* at position 488cs and the break-proximal recipient *YERWdelta22* and break-distal recipient *YERCTy1-1*. An unbiased clone-based assay (as diagrammed in Figure 3A) is similarly used here to nonselectively recover clones after an I-*Sce*I-induced DSB. Position of *URA3* and *LEU2* are indicated. (B) PFGE/Southern analysis of repair clones from two different phenotypic repair classes (Ura^+^Hyg^S^Leu^−^ and Ura^+^Hyg^S^Leu^+^) after DSB at 488cs in purebred and hybrid diploids. (C) Frequencies of *YERCTy1-1* and *YERWdelta22* recipients usage (out of all possible outcomes) in purebred and hybrid diploids after DSB at 488cs on *S. cerevisiae* chromosome V. Usage of the break-distal *YERCTy1-1* recipient is designated a BDR event. Error bars indicate SEM.
(1.74 MB TIF)

Figure S7 Intra-Ty deletion and Ty gene conversion (GC) events after a DSB at RAHScs. BamHI digestion of genomic DNA in agarose plugs followed by PFGE/Southern analysis on 24 Leu^+^Hyg^S^Ura^+^ repair clones generated after a DSB at RAHScs in hybrid (MH3768) and purebred (MH3764) diploids. Intra-Ty deletion (within *RAHS* locus) and Ty GC events have the same repair phenotype (Leu^+^Hyg^S^Ura^+^), but were distinguished by *RAHS* locus size. For Ty GC repair clones, the removal of the small nonhomologous 1.6 kb I-*Sce*cs/*HYG* ends during gene conversion results in a similar size on PFGE/Southern compared to no DSB (first two lanes). For intra-Ty deletion repair clones, the product of deletion within *RAHS* migrates at a smaller size on PFG compared to no DSB.
(1.36 MB TIF)

Table S1 Genotype of yeast strains used in this study.
(0.08 MB DOC)

Table S2 Primers used in this study.
(0.05 MB DOC)

Table S3 Pairwise comparison of global sequence identity between Ty1/Ty2.
(0.52 MB XLS)

Table S4 Pairwise comparison of longest block of perfect identity between Ty1/Ty2.
(0.09 MB XLS)

Table S5 Pairwise comparison of longest block of perfect identity between LTRs. This must be viewed using Excel 2008 or higher due to column and row allowance.
(0.83 MB XLSX)

Table S6 Pairwise comparison of global sequence identity between LTRs. This must be viewed using Excel 2008 or higher due to column and row allowance.
(3.13 MB XLSX)

Table S7 Ranking of chromosome III *RAHS* recipient with all potential Ty1/Ty2 donors.
(0.05 MB XLS)

Table S8 Ranking of *YCRCdelta6* and *YCRCdelta7* recipient with all potential LTR donors.
(0.16 MB XLS)
