# Supplementary figures and images for: Competitive Repair by Naturally Dispersed Repetitive DNA during Non-Allelic Homologous Recombination
Source: PLoS Genet. 2010 Dec 2;6(12):e1001228. doi: 10.1371/journal.pgen.1001228 (PMC2996329; doi:10.1371/journal.pgen.1001228)

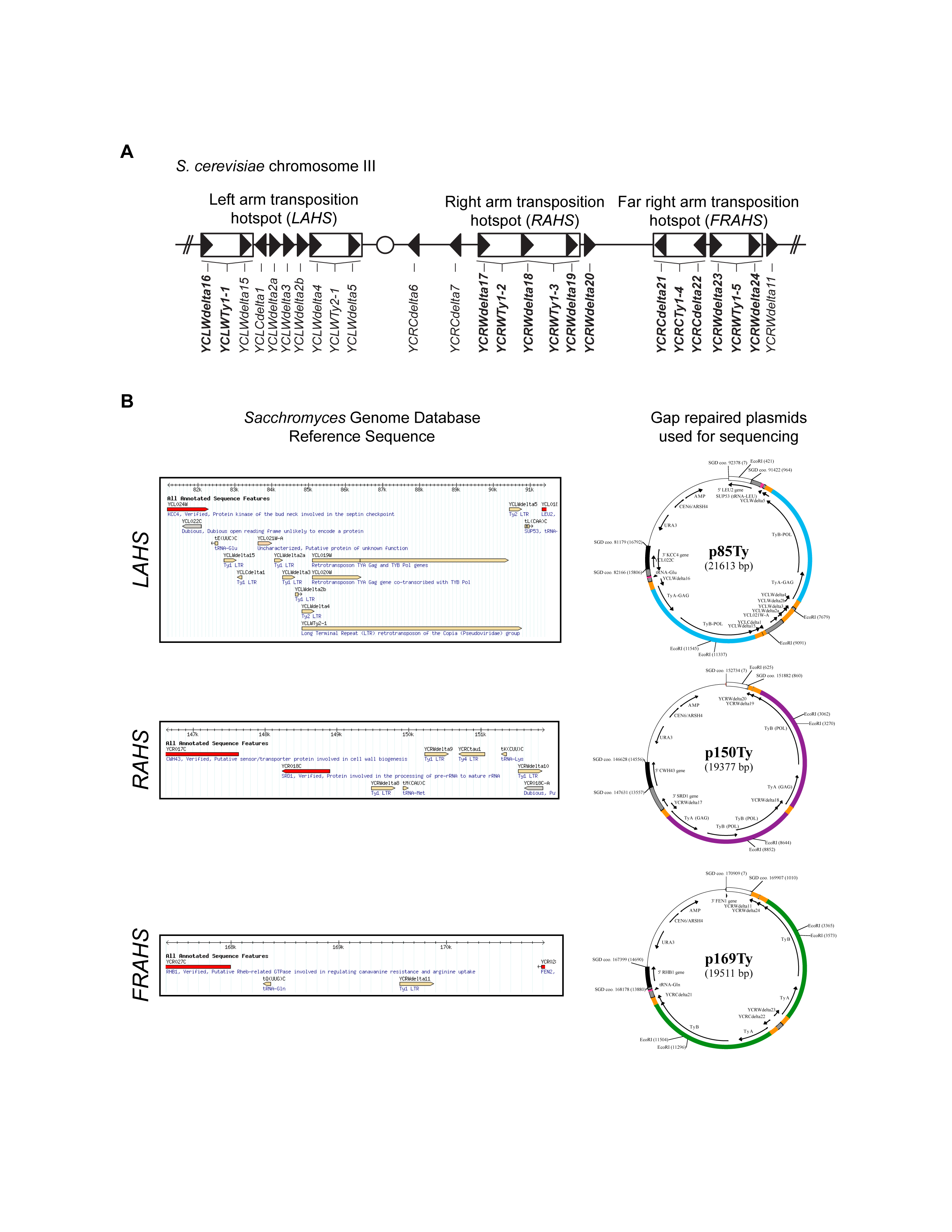

Supplement: Figure S1 — Sequencing of unannotated Ty elements at three Ty clusters on S. cerevisiae chromosome III. (A) Schematic of S. cerevisiae chromosome III showing the Ty configuration of left arm transposition hotspot (LAHS) [Warmington et al 1986], right arm transposition hotspot (RAHS) [Warmington et al 1987], far right arm transposition hotspot (FRAHS) [54] in a standard S288C background. These three loci are herein referred to by their original names in the literature. Unannotated Ty features are given systematic names (bold) in this study according to yeast nomenclature. Full length Tys are shown as open rectangles with triangles (LTRs) inside. Two annotated solo LTRs, YCRCdelta6 and YCRCdelta7, are located between centromere (white circle) and RAHS. (B) Left: Images taken from SGD Gbrowser showing annotated features at LAHS (coordinates 81179–92378), RAHS (coordinates 146628–152734), and FRAHS (coordinates 167399–170909). The reference sequence of chromosome III was based on a composite of four different nonstandard backgrounds [Oliver et al]. Right panel: Yeast clones generated from gap repair of LAHS, RAHS, FRAHS in a standard S288C strain derived from BY4716 [46]. 0.8–1 kb fragments corresponding to the left (black box) and right (white box) of each Ty cluster provided the homology for gap repair. 16,785 bp at LAHS, 14,549 bp at RAHS, and 14,683 bp at FRAHS (pRS316 vector sequence omitted) were deposited into GenBank with accession number GU224294, GU220389, and GU220390, respectively. The deposited sequences include five full length Ty1s and a solo LTR that have not previously been included in any genome-wide Ty sequence analyses. [Warmington JR, Anwar R, Newlon CS, Waring RB, Davies RW, et al. (1986) A ‘hot-spot’ for Ty transposition on the left arm of yeast chromosome III. Nucleic Acids Res 14: 3475–3485.][Warmington JR, Green RP, Newlon CS, Oliver SG (1987) Polymorphisms on the right arm of yeast chromosome III associated with Ty transposition and recombination events. [file pgen.1001228.s001.tif]

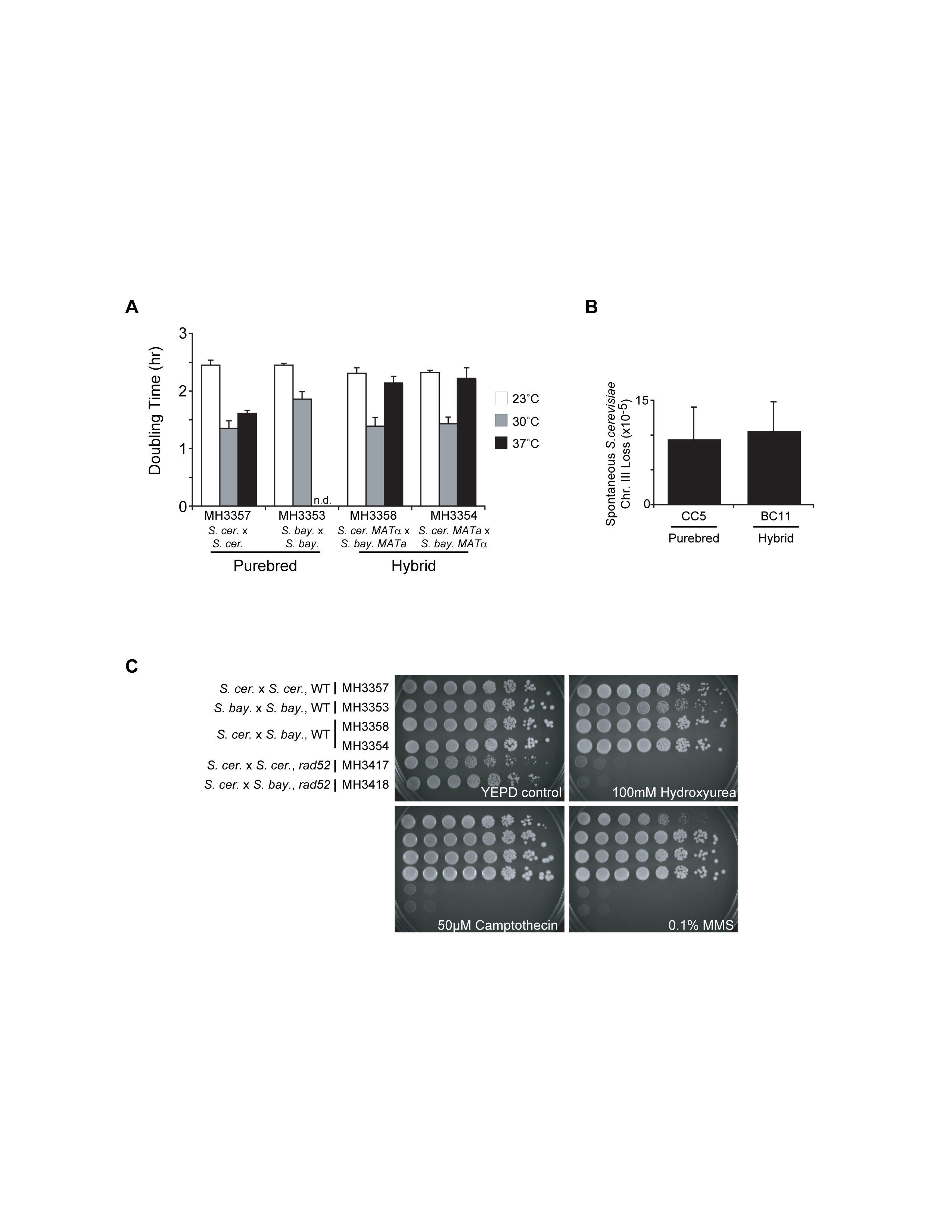

Supplement: Figure S2 — S. cerevisiae/S. bayanus hybrid diploids are competent in DNA maintenance and repair. (A) Doubling time of yeast diploids in YEPD at indicated temperatures. Not determined (n.d.) for S. bayanus purebred diploids at 37°C due to temperature-sensitivity. Error bars indicate SD (n = 3). (B) Frequencies of spontaneous S. cerevisiae chromosome III loss in S. cerevisiae purebred (CC5) and S. cerevisiae/S. bayanus hybrid (BC11). Chromosome III stability genetically monitored by spontaneous loss of both LEU2 (endogenous locus) and URA3 integrated into YCR025C (same disruption used for I-SceI/HYG construct at 163cs). Fresh 23°C overnight YEPD cultures were diluted and plated on 5-FOA, -leu+5-FOA, and YEPD to measure CFU/mL. Plates incubated at 23°C. Loss calculated as [(CFU/mL on 5-FOA) − (CFU/mL on –leu+5-FOA)] / (CFU/mL on YEPD). Error bars indicate SD. At least eight independent cultures assayed for each strain. (C) DNA damage drug sensitivity assayed by a five-fold serial dilutions. Plates incubated for 4 days at 23°C. MMS, methyl methanesulfonate. (1.74 MB TIF) [file pgen.1001228.s002.tif]

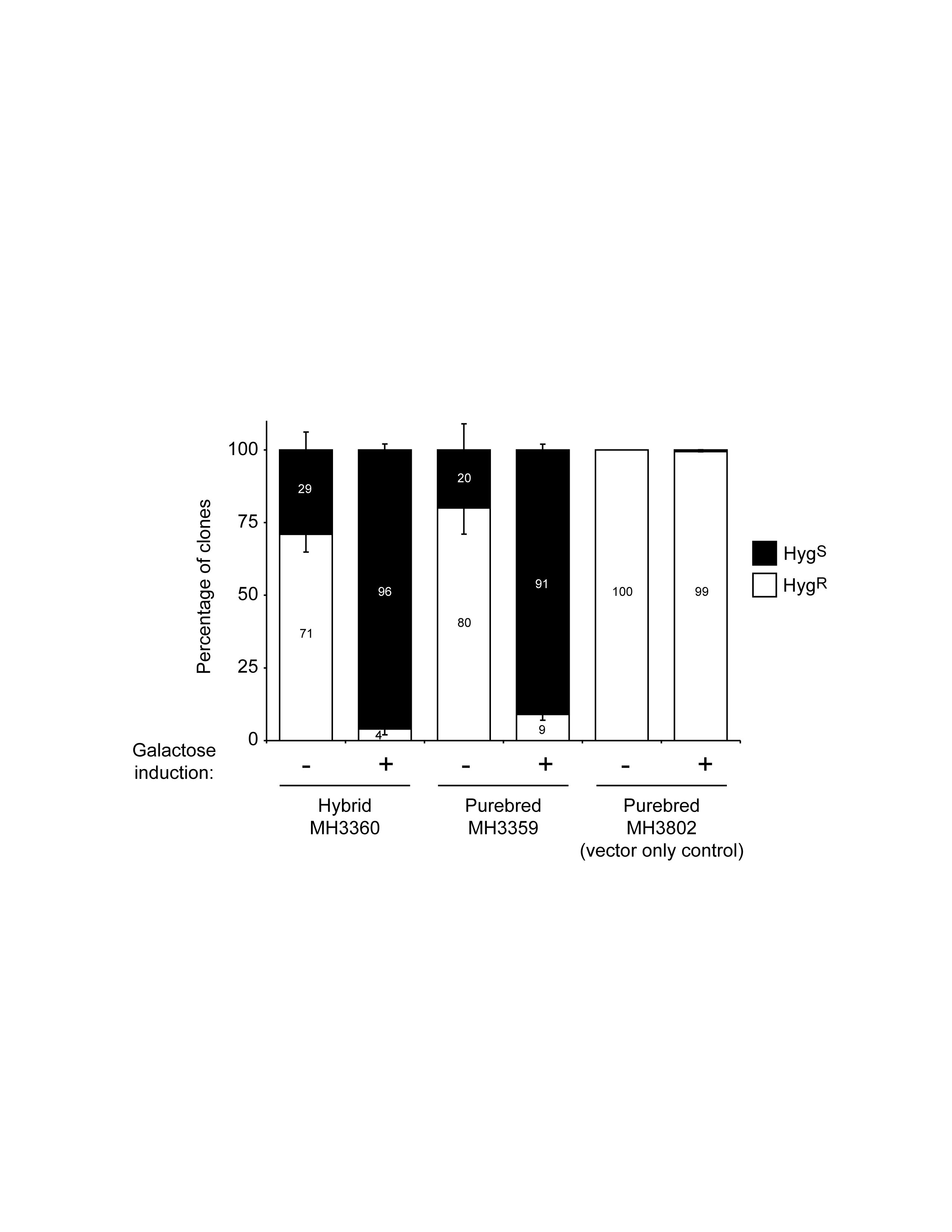

Supplement: Figure S3 — Induction of I-SceI endonuclease leads to Hygromycin-sensitivity. Hygromycin phenotype of clones before (−) and after (+) galactose induction in strains MH3360 and MH3359 (with GALp:I-SceI plasmid construct), and vector only control strain MH3802 (without GALp:I-SceI). Note that the majority of clones are HygR (or no DSB) before galactose addition. The HygS clones observed before induction may be due to leakiness of the galactose promoter during nonrepressive growth. After galactose induction, the small fraction of clones that remain HygR (<10%) may be due to repair through nonhomologous end-joining, inefficient cutting before glucose repression, or loss of the I-SceI expression plasmid. Total number of clones scored before and after galactose induction, respectively, is n = 779 and n = 999 for MH3360, n = 812 and n = 1068 for MH3359, and n = 197 and n = 349 for MH3802. Error bars indicate SD. At least two independent experiments assayed for each strain. (0.65 MB TIF) [file pgen.1001228.s003.tif]

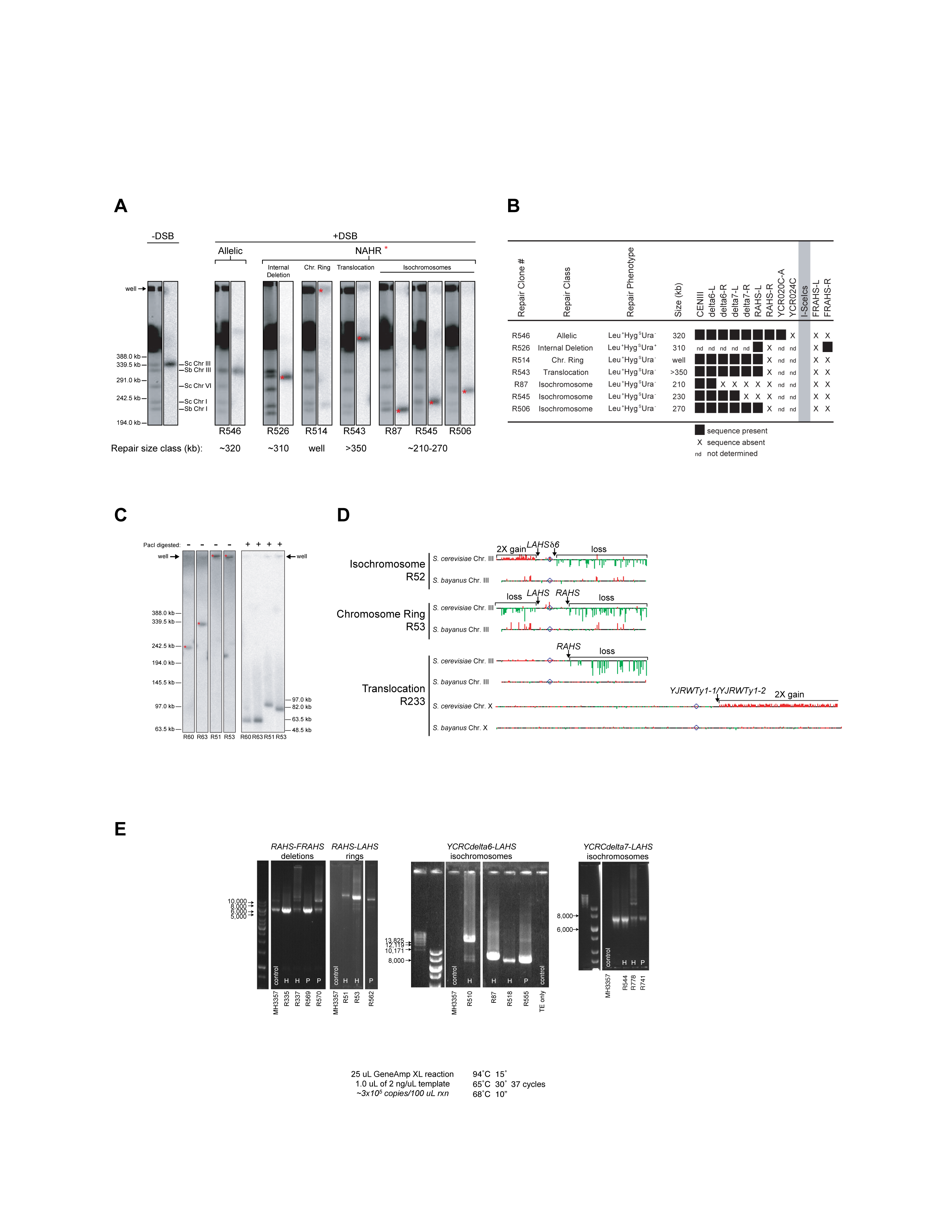

Supplement: Figure S4 — Ty elements mediate rearrangements. (A) Examples of PFG imaged by Ethidium Bromide staining and Southern blotting using LEU2 probe in repair clones from hybrid diploids (MH3360) with or without a DSB. Noted are the size markers (lambda, internal chromosomes) used to determine approximate sizes of bands. Noted below gels is the approximate repair size class. Sizes on PFGE/Southern correlate with rearrangement type and were used to assign rearrangements in hybrids and purebreds diploids. (B) Chart summarizing examples of PCR analysis to determine presence of chromosome III sequences in hybrid repair clones shown in (A). S. cerevisiae chromosome III primer pairs from CENIII to FRAHS identify break-distal Ty recipient locus. For example, in R87 the sequence left of YCRCdelta6 was present (black box) but right of YCRCdelta6 was absent (marked with X), indicating that YCRCdelta6 was at the recombination junction. (C) Release of chromosome rings (R51 and R53) from PFG well by PacI digestion in repair clones generated by hybrid MH3346. Note that strain MH3346 contains an inverted I-SceIcs/HYG construct, but behaves like MH3360. Southern blot using LEU2 probe to PFG with untreated plug samples (four left lanes) and PacI digested plug samples (four right lanes). In untreated R51 and R53, LEU2 probe hybridized to the well with no discrete hybridization in the lane. PacI treated R51 and R53 showed hybridization of a discrete band in the lane. R60 (isochromosome mediated by YCRCdelta7) and R63 (allelic) are also shown for comparison. (D) Examples of aCGH karyoscopes of repair clones from hybrid diploids (MH3346). From the whole genome, only S. cerevisiae chromosome III and relevant chromosomes are shown along with the corresponding S. bayanus homeolog. (E) Examples of the PCR analysis using primers that flank the predicted recombinant junction for the Ty-mediated rearrangements. Bands were amplified using long-range PCR across the junctions for at least one hybrid (H) and one pu [file pgen.1001228.s004.tif]

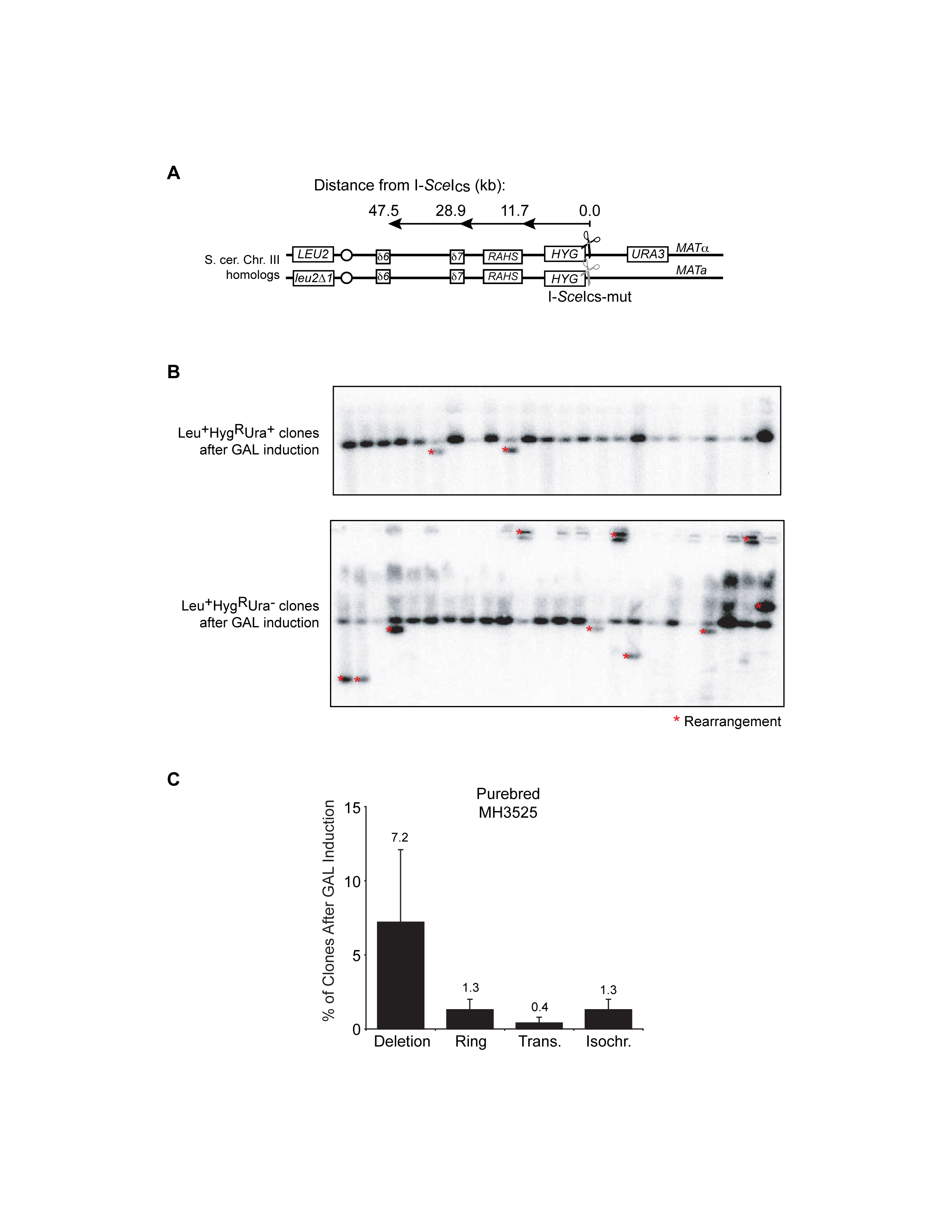

Supplement: Figure S5 — Presence of near perfect identity at the DSB does not prevent break-distal recombination. (A) Map of chromosome III homologs in I-SceIcs/I-SceIcs-mut purebreds (MH3525). MATa homolog contains the same 1.6 kb HYG/I-SceIcs construct at the allelic position of the 163cs, except for a G to A base pair mutation in the I-SceI cut site (mutant 320 in [Monteilhet et al]) that abolishes I-SceI recognition (called I-SceIcs-mut). (B) PFGE/Southern blot using LEU2 probe (hybridizes to both homologs) on Leu+Ura+ and Leu+Ura− random clones after galactose induction. Break-distal recombination using YCRCdelta6 (∂6), YCRCdelta7 (∂7), and RAHS results in Ty-mediated rearrangements, indicated by the repair size class. (C) Frequencies of Ty-mediated rearrangements after galactose induction in purebred MH3525. Note that HYG marker cannot be scored therefore calculated frequencies are likely an underestimate due to a background of uncut cells. For reference, 9% of cells remain uncut (HygR) after galactose induction in wild-type purebred strain MH3359 (see Figure S3). 2116 clones after galactose induction were phenotyped. PFGE/Southern analysis was further performed on 24 Leu+Ura+ and 23 Leu+Ura− random clones (shown in B). Error bars indicate SEM. [Monteilhet C, Perrin A, Thierry A, Colleaux L, Dujon B (1990) Purification and characterization of the in vitro activity of I-Sce I, a novel and highly specific endonuclease encoded by a group I intron. Nucleic Acids Res 18: 1407–1413.] (1.71 MB TIF) [file pgen.1001228.s005.tif]

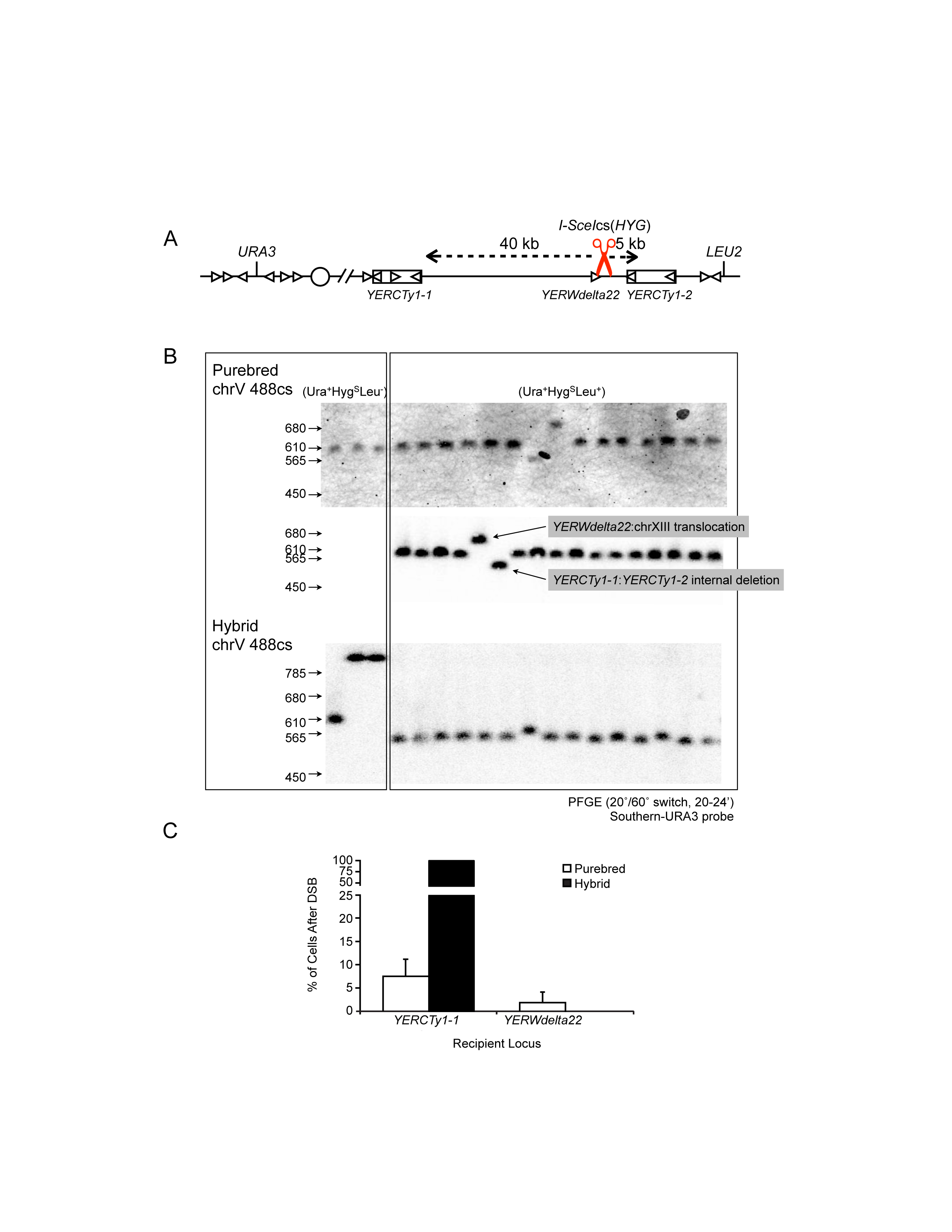

Supplement: Figure S6 — Break-distal recombination (BDR) occurs with an I-SceI-induced DSB on S. cerevisiae chromosome V. (A) Map of S. cerevisiae chromosome V indicating I-SceI cut site (cs) with HYG at position 488cs and the break-proximal recipient YERWdelta22 and break-distal recipient YERCTy1-1. An unbiased clone-based assay (as diagrammed in Figure 3A) is similarly used here to nonselectively recover clones after an I-SceI-induced DSB. Position of URA3 and LEU2 are indicated. (B) PFGE/Southern analysis of repair clones from two different phenotypic repair classes (Ura+HygSLeu− and Ura+HygSLeu+) after DSB at 488cs in purebred and hybrid diploids. (C) Frequencies of YERCTy1-1 and YERWdelta22 recipients usage (out of all possible outcomes) in purebred and hybrid diploids after DSB at 488cs on S. cerevisiae chromosome V. Usage of the break-distal YERCTy1-1 recipient is designated a BDR event. Error bars indicate SEM. (1.74 MB TIF) [file pgen.1001228.s006.tif]

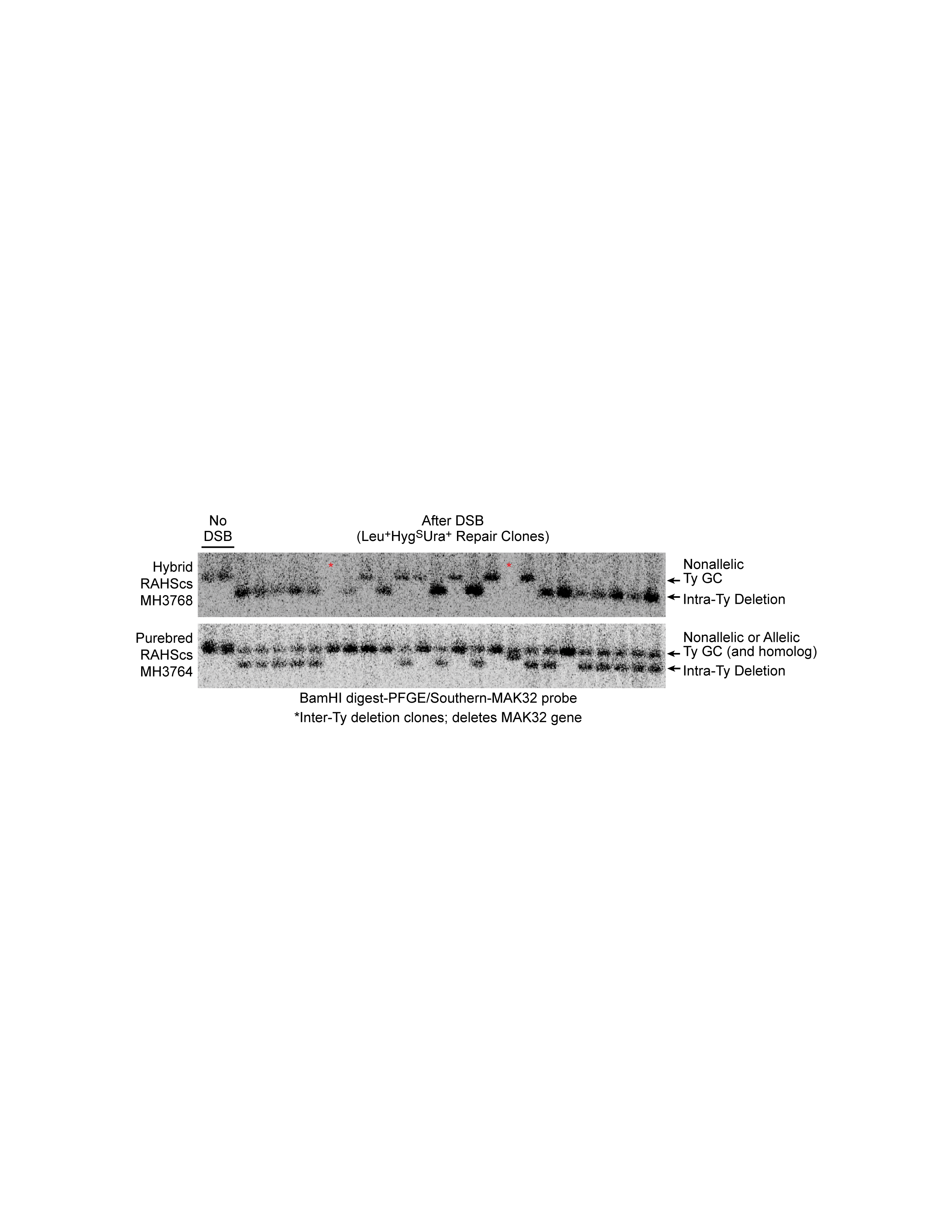

Supplement: Figure S7 — Intra-Ty deletion and Ty gene conversion (GC) events after a DSB at RAHScs. BamHI digestion of genomic DNA in agarose plugs followed by PFGE/Southern analysis on 24 Leu+HygSUra+ repair clones generated after a DSB at RAHScs in hybrid (MH3768) and purebred (MH3764) diploids. Intra-Ty deletion (within RAHS locus) and Ty GC events have the same repair phenotype (Leu+HygSUra+), but were distinguished by RAHS locus size. For Ty GC repair clones, the removal of the small nonhomologous 1.6 kb I-Scecs/HYG ends during gene conversion results in a similar size on PFGE/Southern compared to no DSB (first two lanes). For intra-Ty deletion repair clones, the product of deletion within RAHS migrates at a smaller size on PFG compared to no DSB. (1.36 MB TIF) [file pgen.1001228.s007.tif]
