# Supplementary material for: Competitive Repair by Naturally Dispersed Repetitive DNA during Non-Allelic Homologous Recombination
Source: PLoS Genet. 2010 Dec 2;6(12):e1001228. doi: 10.1371/journal.pgen.1001228 (PMC2996329; doi:10.1371/journal.pgen.1001228)
Supplement: Table S1 — Genotype of yeast strains used in this study. (0.08 MB DOC) [file pgen.1001228.s008.doc]

Supplementary Table S1: Yeast strain list.

***Saccharomyces cerevisiae*** purebred diploids

| **STRAINNAME** | **GENOTYPE** | **PLASMID** |
| --- | --- | --- |
| MH3357 | *LEU2 ycr025c∆::I-SceIcs/HYG bud5∆::URA3 MATα lys2∆0 ho ura3∆0 ade2∆::hisG*  *leu2∆1 MATa LYS2 ho ura3∆0 ade2∆::hisG* | none |
| MH3359 | MH3357 | pMH5 |
| MH3417 | *LEU2 ycr025c∆::I-SceIcs/HYG bud5∆::URA3 MATα lys2∆0 ho ura3∆0 ade2∆::hisG rad52∆::KAN*  *leu2∆1 MATa LYS2 ho ura3∆0 ade2∆::hisG rad52∆::KAN* | none |
| MH3469 | *LEU2 147kb::I-SceIcs/HYG bud5∆::URA3 MATα lys2∆0 ho ura3∆0 ade2∆::hisG*  *leu2∆1 MATa LYS2 ho ura3∆0 ade2∆::hisG* | pMH6 |
| MH3475 | MH3417 | pMH6 |
| MH3502 | *LEU2 ycr025c∆::I-SceIcs/HYG bud5∆::URA3 MATα lys2∆0 ho ura3∆0 rad51∆::KAN ade2∆::hisG*  *leu2∆1 MATa LYS2 ho ura3∆0 rad51∆::KAN ade2∆::hisG* | pMH6 |
| MH3525 | *LEU2 ycr025c∆::HYG/I-SceIcs bud5∆::URA3 MATα lys2∆0 ho ura3∆0 ade2∆::hisG*  *leu2∆1 ycr025c∆::HYG/I-SceIcs-mut MATa LYS2 ho ura3∆0 ade2∆::hisG* | pMH6 |
| MH3764 | *LEU2 RAHS::I-SceIcs/HYG bud5∆::URA3 MATα lys2∆0 ho ura3∆0 ade2∆::hisG*  *leu2∆1 MATa LYS2 ho ura3∆0 ade2∆::hisG* | pMH7 |
| MH3802 | MH3357 | pRS422 |
| FT5633 | *lys2∆0 leu2∆0 MATα ho trp1∆63 URA3 chrV-488::I-SceIcs/HYG chrV-555::LEU2 ::I-SceIcs/HYG met15∆0*  *LYS2 leu2∆1 MATa ho TRP1 ura3∆0 MET15*  *ade2∆::hisG his3∆200*  *ade2∆::hisG HIS3* | pMH7 |
| CC5 | *LEU2 ycr025c∆::URA3 MATα lys2∆0 ho can1 ura3∆0*  *leu2∆1 MATa LYS2 ho CAN1 ura3∆0* | none |

***Saccharomyces bayanus purebred diploids***

| MH3353 | *LEU2 ycr025c∆::I-SceIcs/HYG bud5∆::URA3 MATα lys2-1 ho∆::KAN ura3∆::NAT ade2∆::hisG*  *leu2∆::NAT MATa LYS2 ho∆::KAN ura3∆::NAT ade2∆::hisG* | none |
| --- | --- | --- |

***Saccharomyces cerevisiae (MATα)/Saccharomyces bayanus (MATa)***

hybrid diploids

| MH3346 | *LEU2 ycr025c∆::HYG/I-SceIcs bud5∆::URA3 MATα lys2∆0 ho ura3∆0 ade2∆::hisG*  *leu2∆::NAT MATa LYS2 ho∆::KAN ura3∆::NAT ade2∆::hisG* | pMH5 |
| --- | --- | --- |
| MH3358 | *LEU2 ycr025c∆::I-SceIcs/HYG bud5∆::URA3 MATα lys2∆0 ho ura3∆0 ade2∆::hisG*  *leu2∆::NAT MATa LYS2 ho∆::KAN ura3∆::NAT ade2∆::hisG* | none |
| MH3360 | MH3358 | pMH5 |
| MH3398 | *LAHS∆::NAT LEU2 ycr025c∆::I-SceIcs/HYG bud5∆::URA3 MATα lys2∆0 ho ura3∆0 ade2∆::hisG*  *leu2∆::NAT MATa LYS2 ho∆::KAN ura3∆::NAT ade2∆::hisG* | pMH5 |
| MH3418 | *LEU2 ycr025c∆::I-SceIcs/HYG bud5∆::URA3 MATα lys2∆0 ho ura3∆0 ade2∆::hisG rad52∆::KAN*  *leu2∆::NAT MATa LYS2 ho∆::hisG ura3∆::NAT ade2∆::hisG rad52∆::KAN* | none |
| MH3455 | *LEU2 ycr025c∆::I-SceIcs/HYG bud5∆::URA3 MATα lys2∆0 ho ura3∆0 ade2∆::hisG sgs1∆::KAN*  *leu2∆::NAT MATa LYS2 ho∆::hisG ura3∆::NAT ade2∆::hisG sgs1∆::KAN* | pMH5 |
| MH3471 | *LEU2 147kb::I-SceIcs/HYG bud5∆::URA3 MATα lys2∆0 ho ura3∆0 ade2∆::hisG*  *leu2∆::NAT MATa LYS2 ho∆::hisG ura3∆::NAT ade2∆::hisG* | pMH6 |
| MH3476 | MH3418 | pMH6 |
| MH3507 | *LEU2 ycr025c∆::I-SceIcs/HYG bud5∆::URA3 MATα lys2∆0 ho ura3∆0 rad51∆::KAN ade2∆::hisG*  *leu2∆::NAT MATa LYS2 ho∆::hisG ura3∆::NAT rad51∆::KAN ade2∆::hisG* | pMH6 |
| MH3524 | *LEU2 ycr025c∆::I-SceIcs/HYG FRAHS∆::hisG bud5∆::URA3 MATα lys2∆0 ho ura3∆0 ade2∆::hisG*  *leu2∆::NAT MATa LYS2 ho∆::hisG ura3∆::NAT ade2∆::hisG* | pMH6 |
| MH3551 | *LEU2 151kb::I-SceIcs/HYG FRAHS∆::hisG bud5∆::URA3 MATα lys2∆0 ho ura3∆0 ade2∆::hisG*  *leu2∆::NAT MATa LYS2 ho∆::hisG ura3∆::NAT ade2∆::hisG* | pMH6 |
| MH3572 | MH3524 with different plasmid | pMH5 |
| MH3573 | MH3524 with different plasmid | pMH7 |
| MH3692 | *LEU2 ycr025c∆::I-SceIcs/HYG bud5∆::URA3 MATα lys2∆0 ho msh6∆KAN ura3∆0 ade2∆::hisG*  *leu2∆::NAT MATa LYS2 ho∆::hisG msh6∆KAN ura3∆::NAT ade2∆::hisG* | pMH6 |
| MH3699 | *LEU2 ycr025c∆::I-SceIcs/HYG bud5∆::URA3 MATα lys2∆0 ho ura3∆0 ade2∆::hisG msh2∆KAN*  *leu2∆::NAT MATa LYS2 ho∆::hisG ura3∆::NAT ade2∆::hisG msh2∆KAN* | pMH6 |
| MH3726 | *LEU2 ycr025c∆::I-SceIcs/HYG bud5∆::URA3 MATα lys2∆0 ho rad59∆KAN ura3∆0 ade2∆::hisG*  *leu2∆::NAT MATa LYS2 ho∆::hisG rad59∆KAN ura3∆::NAT ade2∆::hisG* | pMH7 |
| MH3768 | *LEU2 RAHS::I-SceIcs/HYG bud5∆::URA3 MATα lys2∆0 ho ura3∆0 ade2∆::hisG*  *leu2∆::NAT MATa LYS2 ho∆::hisG ura3∆::NAT ade2∆::hisG* | pMH7 |
| FT5626 | *lys2∆0 leu2∆0 MATα ho trp1∆63 URA3 chrV-488::I-SceIcs/HYG chrV-555::LEU2 ::I-SceIcs/HYG*  *LYS2 leu2∆::NAT MATa ho∆hisG TRP1 ura3∆::NAT*  *met15∆0 ade2∆::hisG his3∆200*  *MET15 ade2∆::hisG HIS3* |  |
| BC11 | *LEU2 ycr025c∆::URA3 MATα lys2∆0 ho can1 ura3∆0 .*  *leu2∆::NAT MATa LYS2 ho∆::KAN CAN1 ura3∆::NAT* | none |

***Saccharomyces cerevisiae (MATa) /Saccharomyces bayanus (MATα)***

hybrid diploids

| MH3354 | *LEU2 ycr025C∆::I-SceIcs/HYG bud5∆::URA3 MATα lys2-1 ho∆::KAN ura3∆::NAT ade2∆::hisG*  *leu2∆1 MATa LYS2 ho ura3∆0 ade2∆::hisG* | none |
| --- | --- | --- |
