# Supplementary material for: Competitive Repair by Naturally Dispersed Repetitive DNA during Non-Allelic Homologous Recombination
Source: PLoS Genet. 2010 Dec 2;6(12):e1001228. doi: 10.1371/journal.pgen.1001228 (PMC2996329; doi:10.1371/journal.pgen.1001228)
Supplement: Table S2 — Primers used in this study. (0.05 MB DOC) [file pgen.1001228.s009.doc]

Table S2: Primers used in this study.

| Descriptive Name | Primer Pair |
| --- | --- |
| LEU2 probe | U2-FOR: gtaccggtagtgttagacctg  U2-REV: cgtcgttaaggccgtttctg |
| YCR024C probe | MH-8: gacgtggatagtcggtaacg  MH-9: gcaggtacaccatactagcc |
| MAK32 probe | MH-169: gccgaagaagcagattaacg  MH-170: gaagaccggactgtgaaagg |
| CENIII | PM22: gatcagcgccaaacaatatgg  PM48: aacttccaccagtaaacgtttc |
| delta6-L | MH-90: catttctgccagtagcgaca  MH-91: gggctgtatcccagacttca |
| delta6-R | MH-171: ccgtcctcatgaaatggcta  MH-172: gccctttctgcttcataacg |
| delta7-L | MH-173: aacgaaggaaccgactcaaa  MH-174: ggcggaacggtacataagaa |
| delta7-R | MH-175: gtctaacgctccctttgcag  MH-176: aggaacgtgattttcggaca |
| RAHS-L | MH-167: gaaggcttcagaaacgaacg  MH-168: ccttctttgcagctttggtc |
| RAHS-R | MH-178: cggtcatcggaatctcagtt  MH-179: cagcccttttcttccaggta |
| YCR020C-A | MH-96: gctgactaggcccatcattc  MH-97: gcgaagtgcatgagttctga |
| YCR024C | same as YCR024C probe |
| FRAHS-L | MH-162: gcatcaagccccagagttcc  MH-163: tacgaggcataatactgtgc |
| FRAHS-R | MH-180: gttctcactgacgctgcttg  MH-181: ctagctccggtcggaatttt |
| LAHS∆::NAT | MH-147: actcaactgttccaataaaatctgcaatttaaattaattattctgaaaaatcaggggcatgatgtgact  MH-148: tgttgagaaatagagtataattatcctataaatataacgtttttgaacacagctcgttttcgacactggat |
| FRAHS∆-left | MH-241: aacgctactggtggtttctct  paired with MH-162 |
| FRAHS∆-middle | MH-239: ctggcagaagcgtcttgttaatacttatagagaaaccaccagtagcgttagggaacaaaagctgggtacc  MH-240: gaatttaatatggctacagcaagatgttctgaaggatgatcgtgatctgttcaggtgctatagggcgaattggagct |
| FRAHS∆-right | MH-242: tcacgatcatccttcagaaca  paired with MH-181 |
| 147cs | DK-43: ctttgattgtaaaaaacaacattaaaaagaactgcaaacctgaaccaagaatattacgctagggataacagggtaatatagcgtcaggggcatgatgtgact  DK-44: gtttaagtgaacttctacaagttgtacctcatttctcggttttgaaattaagagctcgttttcgacactggat |
| RAHScs | MH-337: ccaccggtggttacgctagggataacagggtaatatagcgtcaggggcatgatgtgact  MH-338: catcgatgagctcgttttcgacactggat |
| 151cs | MH-246: ccgtgtttgaatgttgatctgaaaacaaaaattaatctctcgtacttattttacgctagggataacagggtaatatagcgtcaggggcatgatgtgact  MH-247: accatctaacaagaaaatcatttggaattttgagaaaatacaatatccatagctcgttttcgacactggat |
| 163cs | MH-54: atgtcgtcatttggacatcgagcatttttcagctgttgtcctttactaagctcgttttcgacactggat  MH-55: acgcagtgacaagtctatattgacatctttctgctttctgctttctgtttacgctagggataacagggtaatatagcgtcaggggcatgatgtgact |
| I-SceIcs-mut | MH-243: atgtcgtcatttggacatcgagcatttttcagctgttgtcctttactattacgctagggataacaaggtaatatagcgtcaggggcatgatgtgact  MH-45: acgcagtgacaagtctatattgacatctttctgctttctgctttctgtagctcgttttcgacactggat |
| 488cs (chrV) | FT-55: aatagcccttcaccttacattgcaccactggttgaacagtccatttttaattacgctagggataacagggtaatatagcgtcaggggcatgatgtgact  FT-56: ctcgtacaggtttacatgccccattataccaataacaaaatgggagagatagctcgttttcgacactggat |
| Deletion  (RAHS-FRAHS) | FT-114: tccctgttttggtcgggtccactacgc  FT-118: cgcgcgagaaagagtccagagacaaagg |
| Ring  (RAHS-LAHS) | FT-122: accacccacagcacctaacaaaacggc  paired with FT-114 |
| Isochromosome  (YCRCdelta7-LAHS) | FT-124: aggtttcaaccgcgggttcagtagatacg  FT-123: tcaaaaagcgcagagcccacactgg |
| Isochromosome  (YCRCdelta6-LAHS) | FT-119: tgaaaaatcccggcagaacagcgcc  paired with FT-123 |
